# Supplementary material for: Attitudes towards a programme of risk assessment and stratified management for ovarian cancer: a focus group study of UK South Asians’ perspectives
Source: BMJ Open. 2018 Jul 18;8(7):e021782. doi: 10.1136/bmjopen-2018-021782 (PMC6059306; doi:10.1136/bmjopen-2018-021782)
Supplement: Supplementary data [file bmjopen-2018-021782supp003.pdf]

Ovarian cancer risk

- The lifetime risk of a women in the UK getting ovarian cancer is about 2%.
- This means around 1 in 50 women will develop ovarian cancer at some point in their life.

Ovarian cancer risk

- We don’t know the cause of most ovarian cancers.
- We know some of the ‘**risk factors’ –** these are things that may increase the chances of developing cancer.
- Having a cancer risk factor doesn’t mean that a person will definitely get cancer – just as not having it doesn’t mean that they won’t.

Genes

- Genes carry the biological information passed from parent to child.
- Some genes are known to increase the risk of ovarian cancer.
- Changes (mutations) in certain genes are known to increase the risk of cancer.

Ovarian cancer genes

- Mutations in two genes – called BRCA1 and BRCA2 – increase the risk of ovarian cancer.
- BRCA1 and BRCA2 mutations are very rare but family members who inherit them have a much greater risk of ovarian cancer.
- Scientists can also identify other relevant gene mutations involved.

Testing for genetic risk

- It is possible to test for BRCA1 and BRCA2 gene mutations.
- Genetic testing involves a blood test.
- Genetic material (DNA) is taken from the blood cells to test for mutations.
- At present genetic testing for ovarian cancer is not available on the NHS except for women with a strong family history of cancer.

Importance of family history of cancer

- Families with a strong 'family history' of ovarian cancer are more likely to carry the gene mutations.
- A strong family history means:
  - – Two close relatives (mother, sister, daughter) with ovarian cancer
  - – One close relative with ovarian cancer, and, on the same side of the family:
    - One close relative who had breast cancer before age 50
    - Two close relatives who had breast cancer before age 60
    - Three close relatives who had bowel or womb (uterus) cancer

PROMISE research programme

- Genetic testing + personal and lifestyle information = risk
- Women who agree to this will be grouped as being at **high**, **intermediate** or **low** risk for ovarian cancer.

PROMISE continued…

- The doctor would then discuss different risk management options:

➢ Low risk: symptom awareness information.

➢ Intermediate risk: screening, or surgery depending on age.

➢ High risk: surgery, or screening if not ready to have surgery.
