# Supplementary material for: Attitudes towards a programme of risk assessment and stratified management for ovarian cancer: a focus group study of UK South Asians’ perspectives
Source: BMJ Open. 2018 Jul 18;8(7):e021782. doi: 10.1136/bmjopen-2018-021782 (PMC6059306; doi:10.1136/bmjopen-2018-021782)
Supplement: Supplementary data [file bmjopen-2018-021782supp002.pdf]

**Title: Attitudes towards a programme of risk assessment and stratified management for ovarian cancer: A focus group study of UK South Asians' perspectives**

**Focus Group Discussion Guide (Men)**

1. Introduction

- Welcome and thanks for taking part
- Introduction and purpose of the study
- Confidentiality (agree that the discussion should be confidential among respondents)
- Timing (up to 1 ½ hours)
- Reminder of audio-recording
- Anonymity in report writing etc.
- Ground rules – respect different opinions, keep mobile phones off or on silent
- No right/wrong answers – hoping for a range of views
- Participants introduce themselves

2. General awareness/attitudes towards ovarian cancer risk

- I'd like to start by discussing what you know about ovarian cancer?
    - Prompts: What do you know about how common ovarian cancer is/symptoms/ causes/ risk factors/reducing risk?

***Focus group participants presented with slides on genetic risk.**

3. Opinions on genetic information

*We are working on a project where we will be inviting women to have genetic testing for ovarian cancer risk, regardless of whether they have a family history of cancer.*

- Does genetic cancer risk information make sense to you?
    - What did you know about genetic testing for cancer risk before coming to this discussion today?
    - Does the link between genetics and risk of ovarian cancer make sense?
- How would you feel about female members of your family (your wife/ sister/ mother/ daughter) having genetic testing and receiving test results on ovarian cancer risk?
    - Prompt: pros/cons? Culturally acceptable? Religiously acceptable?
    - Prompt: What impact could it have on you/your family?

4. Opinions of risk stratification approach and possible risk management options

*In our project, women will be told whether they have a low, intermediate or high risk for ovarian cancer, based on a combination of their genetic risk and other risk factors. Identifying other risk factors is done using questions about family history, lifestyle and health information. We expect that most of the women will have a low risk result, fewer will have an intermediate risk, and fewer still will have a high risk. Being at low risk would not mean that the women have no risk of developing ovarian cancer.*

*Depending on their risk level, women would be offered different interventions. Women at low risk would receive information telling them that they are low risk and don't need further monitoring, they would also be given information about symptoms of ovarian cancer to be aware of. Women at intermediate risk would be offered screening every four months involving a blood test to check for levels of a biomarker and a yearly ultrasound scan. Preventative surgery, involving the removal of the*

*ovaries, would be discussed as an option depending on the characteristics of the woman (such as age, their family history of cancer etc). For high risk women, preventative surgery would be offered as a primary option, but screening would also be discussed with these patients.*

- What do you think about this idea?
  - Prompt: Pros and cons?
  - Prompt: Culturally acceptable? Religiously acceptable?
  - Prompt: What do you think about the idea of offering different care/options to women depending on their level of risk?
  - Prompt: What do you think about the risk management options (information/screening/surgery)?
  - Thinking about wife/mother/sister: what impact do you think a high/ intermediate/ low risk would have for them/the family?

*Much of what we know about ovarian cancer risk due to inherited genetics comes from research with white women of European descent. Whilst there is no evidence to suggest that this risk differs between ethnic groups, we don't know for certain. It is possible that the estimated cancer risk based on previous research may be less accurate for other ethnic groups.*

- How might this information impact on whether or not you would support or encourage female family members' decision to have their cancer risk estimated from genetic and other information?

- How might this programme of genetic testing and risk management be provided in a way that would be acceptable and accessible?
  - What would be important about how the service is provided?
  - Barriers/facilitators?

<u>5. Final comments</u>

- Is there anything else that you'd like to talk about that you think might be relevant?

<u>6. Debrief and thank participants</u>
