# Supplementary material for: Attitudes towards a programme of risk assessment and stratified management for ovarian cancer: a focus group study of UK South Asians’ perspectives
Source: BMJ Open. 2018 Jul 18;8(7):e021782. doi: 10.1136/bmjopen-2018-021782 (PMC6059306; doi:10.1136/bmjopen-2018-021782)
Supplement: Author's manuscript [file bmjopen-2018-021782.draft_revisions.pdf]

BMJ Open is committed to open peer review. As part of this commitment we make the peer review history of every article we publish publicly available.

When an article is published we post the peer reviewers' comments and the authors' responses online. We also post the versions of the paper that were used during peer review. These are the versions that the peer review comments apply to.

The versions of the paper that follow are the versions that were submitted during the peer review process. They are not the versions of record or the final published versions. They should not be cited or distributed as the published version of this manuscript.

BMJ Open is an open access journal and the full, final, typeset and author-corrected version of record of the manuscript is available on our site with no access controls, subscription charges or pay-per-view fees (http://bmjopen.bmj.com).

If you have any questions on BMJ Open's open peer review process please email info.bmjopen@bmj.com

BMJ Open

Attitudes towards a programme of risk assessment and stratified management for ovarian cancer: A focus group study of UK South Asians' perspectives

Journal:	*BMJ Open*
Manuscript ID	bmjopen-2018-021782
Article Type:	Research
Date Submitted by the Author:	18-Jan-2018
Complete List of Authors:	Hann, Katie; University College London; Royal Holloway, University of London, Health Psychology Research Unit<br>Ali, Nasreen; University of Bedfordshire Faculty of Health and Social Sciences, Institute for Health Research<br>Gessler, Sue; University College London Hospitals NHS Foundation Trust, ; UCL Institute for Women's Health and NIHR University College London Hospitals Biomedical Research Centre, Department of Women's Cancer<br>Fraser, Lindsay; Institute for Women's Health, Department of Women's Cancer<br>Side, Lucy; University College London; University Hospital Southampton<br>Waller, Jo; UCL, Epidemiology and Public Health<br>Sanderson, Saskia; University College London<br>Lanceley, Anne; UCL, Department of Women's Cancer
Keywords:	ovarian cancer, genetic testing, risk assessment, risk stratification, Risk management < HEALTH SERVICES ADMINISTRATION & MANAGEMENT

SCHOLARONE™
Manuscripts

Title: Attitudes towards a programme of risk assessment and stratified management for ovarian cancer: A focus group study of UK South Asians' perspectives

Running title: Attitudes to ovarian cancer risk stratification.

Hann, K. E J.,[1,2] Ali, N.,[3] Gessler, S.,[1] Fraser, L.,[1] Side, L.,[1,4] Waller, J.,[5] Sanderson, S. C.,[5,6] & Lanceley, A.[1] for the PROMISE study team.

[1]Dept. of Women's Cancer, EGA UCL Institute for Women's Health, University College London, London, UK, WC1E 6AU.

[2]Health Psychology Research Unit, Royal Holloway, University of London, Egham, Surrey, UK, TW20 0EX.

[3]Institute for Health Research, University of Bedfordshire, Luton, Bedfordshire, UK, LU2 8LE.

[4]Clinical Genetics, University Hospital Southampton NHS Foundation Trust, Southampton, UK, SO16 5YA

[5]Dept. Behavioural Science and Health, Institute of Epidemiology and Health Care, University College London, London, UK, WC1E 6BT.

[6] Great Ormond Street Hospital, London, UK, WC1N 3JH.

Corresponding Author:

Anne Lanceley, PhD

237c

Medical School Building

74 Huntley Street

London, WC1E 6AU

Email: a.lanceley@ucl.ac.uk

Phone: +4420 7679 6807

For peer review only - http://bmjopen.bmj.com/site/about/guidelines.xhtml

For peer review only

Abstract

Objective: Population-based risk assessment, using genetic testing to identify those at high, intermediate and low risk, and the provision of appropriate risk management could lead to prevention, early detection and improved clinical management of ovarian cancer (OC). This study explored attitudes of South Asian (SA) women and men in the UK regarding such a programme with the aim of identifying how best to implement such a programme to minimise distress and maximise uptake.

Design: Semi-structured qualitative focus group discussions.

Setting: Community centres across North London and Luton.

Participants: 49 women and 13 men who identified as SA (Indian, Pakistani or Bangladeshi).

Methods: 7 community based focus groups were held. Group discussions were transcribed verbatim, coded and analysed thematically.

Results: Awareness and knowledge of OC symptoms and specific risk factors was low. The programme was acceptable to most participants and attitudes to it were generally positive. Participants' main concerns related to receiving a high risk result following the genetic test. Younger women may be more cautious of genetic testing, screening, or risk-reducing surgery due to the importance of marriage and child bearing in their SA cultures.

Conclusions: A crucial first step to enable implementation of population based genetic risk assessment and management in OC is to raise awareness of OC within SA communities. It will be important to engage with the SA community early on in programme implementation to address their specific concerns and to ensure culturally tailored decision support.

Strengths and limitations of this study

- This is the first study to explore the attitudes of a UK ethnic minority group towards population-based risk assessment and stratified management for ovarian cancer.
- The study explored the attitudes of both women and men.

For peer review only - http://bmjopen.bmj.com/site/about/guidelines.xhtml

- Opinions solicited during the focus groups were directly related to information provided about population-based risk assessment and stratified management for ovarian cancer and this may have limited responses.
- Two female researchers facilitated all the focus groups including those with men, this may have influenced the findings.

Key words: Ovarian cancer, genetic testing, risk assessment, risk stratification, risk management, South Asian

For peer review only

For peer review only - http://bmjopen.bmj.com/site/about/guidelines.xhtml

Introduction

Ovarian cancer (OC) is the sixth most common cancer amongst UK women[1]. Due to the non-specific symptoms associated with this cancer, diagnosis is usually at a late stage when prognosis is poor[2]. Earlier detection of OC could help to save lives and this has fuelled voluntary sector demands for research to investigate approaches for prevention and earlier diagnosis [3]. A definitive ongoing trial investigating screening for OC in postmenopausal women has shown this to be sensitive and feasible[4 5], but to date without a significant mortality benefit[5,6]. Nevertheless, a stage shift at diagnosis has been evidenced and for high risk women who are not ready to have risk-reducing surgery [5], screening could be an interim option.

Mutations of *BRCA1* and *BRCA2* genes considerably increase an individual's risk of OC;[7] combined with non-genetic information (e.g. family history of cancer, age, and lifestyle factors), this genetic information can be used to estimate a woman's risk. Following risk assessment, risk stratified management could benefit patients by identifying those at high risk and in most need of management, whilst avoiding over-investigation of those at lowest risk[8 9]. A current programme of research, Predicting Risk of Ovarian Malignancies, Improved Screening and Early Detection (PROMISE, https://eveappeal.org.uk/our-research/our-research-programmes/promise-2016/), involves a feasibility trial to investigate whether stratified OC risk management is acceptable to women in the UK general population. In this programme, women will be provided with an estimate of their OC risk and stratified as low, intermediate or high risk. Those with the lowest risk will be provided with information on OC. Screening, or surgery to remove the ovaries, will be offered to those at intermediate and high risk. In the future, equivalent programmes could be rolled out for other cancers[10].

Positive attitudes to the PROMISE programme were reported amongst women in the general [11] and in high OC risk populations[12], but study samples did not reflect the diverse UK population of whom

For peer review only - http://bmjopen.bmj.com/site/about/guidelines.xhtml

approximately 5.3% are South Asian (SA) (Pakistani, Bangladeshi and Indian)[3]. A key concern for any public health programme is its inclusivity, yet little is known about UK ethnic minority peoples' awareness and attitudes toward genetic testing for cancer risk [14]. The few studies that have explored delayed use of genetic services among UK minority groups identify low awareness of their availability, language barriers, and unwillingness to discuss cancer due to stigma and fear as contributing factors[15 16].

This study aimed to explore SA women's and men's attitudes to the idea of population-based genetic testing and risk stratified management of OC, and to identify factors which may influence participation.

Methods

Methodological approach

The study took a constructionist perspective in which meaning and experience are considered to be socially produced and reproduced rather than as immutable individual characteristics[17]. It used a qualitative research design of focus groups to explore existing knowledge of OC and views of the novel population-based risk management intervention. Focus groups are well suited to exploration of public health topics and are a good way of identifying community norms and cultural values[18]. Structured discussion within the groups provided an opportunity for participants to question each other and reflect upon and challenge one another's views. Thematic analysis of these data was undertaken.

Patient/public involvement

Patients and the public were involved as project steering group members in the design of the overall PROMISE programme and the health behaviour work-stream within which this study was delivered.

For peer review only - http://bmjopen.bmj.com/site/about/guidelines.xhtml

For peer review only

Members of the SA community also contributed by pilot testing our presentation materials for the focus groups.

Setting

Participants were recruited from the North London Boroughs of Brent, Newham and Tower Hamlets and Luton areas which have large SA settler communities. Groups were conducted in suitable local community venues between November 2016 and April 2017. Approval to conduct the study was granted by the UCL Research Ethics Committee (project ID: 8053/003).

Participants

Purposive sampling was used to include only individuals ≥18 years old, who self-identified as being of South Asian ethnicity (Indian, Pakistani, Bangladeshi) and to include a wide spread of ages. At least some conversational English language was needed to take part. Men were included in the study as they may play a role in supporting and advising female family members' healthcare decisions. Women were excluded if they had (a) a diagnosis of OC and/or (b) previously had genetic testing to find out about personal cancer risk.

SA women and men were introduced to the study by local community centre staff (N= 53), and through poster and leaflet advertisement at community centres and by a local women's health organisation (N= 9). The few eligible individuals who contacted the research team directly by phone or email were sent the study information and had an opportunity to ask questions. We aimed to obtain a broad range of views and continued to recruit until we achieved data saturation,[19] when no new views were being expressed. Of those who agreed to participate, 2 withdrew due to sickness. Participants received a £20 gift voucher and travel costs.

For peer review only - http://bmjopen.bmj.com/site/about/guidelines.xhtml

Data collection

Seven focus group discussions were held at community centres: five with women (n=12, n=8, n=9, n=11, n=9) and two with men (n=7, n=6). Each discussion lasted approximately 75 minutes. Groups were facilitated by NA, a SA multilingual senior qualitative researcher, and KH, a research assistant with a Masters level qualification, acting as moderator and note-taker alternatively. A semi-structured discussion guide developed from previous work [11] and the literature and which comprised open-ended, none-directive questions, was used (See Additional Files 1 & 2). These aimed to facilitate discussion and elicit participant views.

At the start of each focus group KH and NA introduced themselves briefly (name, job, associated university), stated the study's purpose and confirmed what participation involved. Intra-group confidentiality, audio–recording and study report confidentiality were highlighted with an opportunity to ask questions. Participants gave written consent and each completed a demographic questionnaire.

To open the discussion participants were invited to share their current awareness and knowledge of OC. Essential information concerning OC, including the increased risk amongst those with *BRCA1/2* gene mutations, the possibility to test for these and the PROMISE programme's proposal to offer OC risk-stratified management (see Additional file 3), was then given in a short slide presentation and hand-out. It was also explained that OC risk information from genetic testing could be less accurate for women of SA ethnicity as most research has been carried out with women of European descent[20]. The presentation text was designed to be understood by participants irrespective of educational attainment and was pilot tested with SA women for comprehension.

Two groups were conducted solely in English, and five in multiple languages including English, Urdu, Hindi, Punjabi, Pahari and Bengali. In two groups involving Bangladeshi women, those fluent in

For peer review only - http://bmjopen.bmj.com/site/about/guidelines.xhtml

English assisted their peers so that everyone understood the language used. In one group which included Bangladeshi men a woman acted as a translator to help a few participants take part in the discussion.

Analysis

Group discussions were audio recorded, translated into English if necessary, transcribed verbatim by a professional multi-lingual transcription service, and checked against the recordings for accuracy by KH and NA. The data was analysed thematically [21] using QSR International's NVivo 10 Software (2012). KH read and re-read the transcripts and generated initial codes. AL and NA also read the transcripts to identify any divergent cases, and initial codes were refined after discussion. Themes were identified deductively, guided by the discussion topics, and inductively, as they emerged from the data. KH analysed all seven transcripts and an independent researcher (SG) coded two transcripts. KH and SG met to confirm any divergent cases and discuss any disagreements in coding until a consensus was reached. This paper follows the consolidated criteria for reporting qualitative studies (COREQ) [22].

Findings

A total of 49 women and 13 men took part. Demographic characteristics of participants are presented in Table 1. Five themes were identified: participants' awareness and knowledge of OC and genetic risk; attitudes towards genetic testing and finding out about OC risk; attitudes towards risk stratified management; family, culture and religion; and accessing services.

1. Awareness and knowledge of OC and genetic risk

The term ovary/ovaries was not familiar to many participants. In all groups there was some confusion over the ovaries, what they are and where they are located in the body. Some participants had difficulty distinguishing between the ovaries and the womb and this was reflected in some Urdu

For peer review only - http://bmjopen.bmj.com/site/about/guidelines.xhtml

speakers using the word ‘bacha daani’ (womb) and ‘undah daani’ (ovaries) interchangeably. Most participants were aware of the UK’s common cancers mentioning breast, prostate, and lung, as well as cervical cancer, and correctly named some of the main risk factors for these. However, the majority had not come across OC and were unaware of the risk factors and main symptoms of the disease.

Ovarian cancer.....no one’s heard of it

FG4, woman, Luton.

…I’m quite aware, I do pick up leaflets and read but I don't think I've come across ovarian cancer, not on TV, not on any sort of media, not on the train, nothing.

FG3, woman, London.

A few women incorrectly believed that use of hormone replacement therapy or the contraceptive pill would increase a woman’s risk of OC. Older age was infrequently reported as a risk factor and few participants spontaneously spoke about family history or genetic risk. A minority of participants who demonstrated awareness of OC explained that this was due to either having researched the topic online prior to the group discussion (n=1) or from experience of a relative with OC (n=1).

Likely due to the lack of awareness, the women had not considered their risk of OC. Some indicated that they did not generally think about their personal risk of cancer. When asked, most acknowledged that they would have some risk of OC, although whether they perceived this to be the same, lower, or higher than others in the general population varied within and between the groups.

Other populations, I think it’s the same?

FG1, woman, London.

It’s higher in Asian

For peer review only - http://bmjopen.bmj.com/site/about/guidelines.xhtml

But tell me this, I have never heard of any Asian person with ovarian cancer

FG4, woman, Luton.

Most participants had not heard of genetic testing for cancer risk and those that had did not know about the specific *BRCA1/2* genes. After participants had been informed about genetic testing within the group it became apparent that some had difficulty understanding that a) the test would provide information about a person's risk rather than a cancer diagnosis, and b) being at risk did not mean that they would definitely develop cancer.

2. Attitudes toward genetic testing and finding out about OC risk

Based on our presentation of genetic risk and genetic testing for OC, in discussion most participants initially expressed positive views. They felt they would benefit from knowing if they were at increased risk because they could take steps to manage their individual risk.

So you are aware of it, and you know how to prevent it, getting information, what are the risks, and how to do your daily activity, your daily lifestyle, maybe that can change …

FG2, woman, London.

The majority of women indicated that they would accept genetic testing, and several men said that they would encourage female family members to have testing if it were offered, although they acknowledged that ultimately it would be the individual's decision. Many participants remained positive about genetic testing even after being told that risk information could potentially be less accurate for ethnic minorities. Participants said there were no cultural or religious prohibitions on genetic testing for cancer risk and these aspects of the discussion prompted some Muslim participants to speak of the positive influence that religion has on maintaining good health. In one group, participants referred specifically to the Imam (Muslim religious scholar), and his role in providing guidance to the community on health practices.

For peer review only - http://bmjopen.bmj.com/site/about/guidelines.xhtml

Your religion wants you to look after yourself.

FG1, woman, London.

The main concerns voiced about genetic testing for OC risk related to experiencing worry between the blood test and receiving the result, and fear regarding the psychological impact of a high risk result and what such a result would lead to if received:

"Until you know the outcome, your brain will be working overtime"

FG4, woman, Luton.

Participants were not fearful of providing blood for the genetic test and some indicated that whilst they were positive about genetic testing others might be wary since they may be unfamiliar with OC, genetic testing, and fearful of cancer per se.

"Negative side could be some people, maybe my mother, wouldn't wanna go to that test, maybe she would be scared, even if she doesn't have cancer…."

FG2, woman, London.

Other negative aspects, such as the cost to the government or concerns that the test or 'diagnosis' could be wrong or inconclusive, were infrequently discussed.

3. Attitudes towards risk stratified management

Participants endorsed the risk stratified management approach and accepted the information that there would be clear options for women at each level of risk. No concerns were expressed about receiving different treatment based on level of risk.

…you will be able to find out what you have got and according to that you can prevent your, you know, things as well, if you want to go for like a screening or for a minor surgery or whatever it is, it's good to know what you have.

FG1, woman, London.

For peer review only - http://bmjopen.bmj.com/site/about/guidelines.xhtml

For peer review only

The screening component of risk-stratified management was generally endorsed, and participants felt that within their SA communities participation in current UK cancer screening programmes was gaining traction:

> *Well at least it's something for your health, good health*
>
> FG3, woman, London.
>
> *It's best to take a test, best to take a test not to get to that stage, isn't it?*
>
> FG7, man, Luton.

However, participants talked about there still being some within the SA community who do not accept cancer screening per se. A few participants, mostly men, suggested that some women may not see the need to attend screening in the absence of symptoms. Reluctance to attend screening was also attributed to fear and issues of body privacy and shyness. Women owned that exposing their body to "someone else" could be distressing, irrespective of whether the person was a health care professional:

> *What she's saying is that first, Muslims were a bit scared and they wouldn't get checks done. They thought that maybe someone else would see them...*
>
> FG4, woman, Luton.

Attitudes towards risk-reducing surgery were mixed. Whilst surgery was acceptable if it was deemed necessary by doctors, men and women said that women would not want surgery unless they had already had children and were of an older age.

> *It depends on age because, any lady who is 50 years up, that time is high risk, she needs to remove that, but 25 years, 30 years, any lady, she has still option for children, so she can't do that thing*
>
> FG2, woman, London.

For peer review only - http://bmjopen.bmj.com/site/about/guidelines.xhtml

Some men did not agree with risk-reducing surgery and believed that women would not accept it until cancer was diagnosed especially since ‘high risk’ did not mean the woman would definitely develop OC.

Until such a time that a person is diagnosed with cancer, I don’t think they will have their ovaries removed.

FG5, man, Luton.

4. Family, culture and religion

The majority of women anticipated that their husbands and immediate families would support them if they had genetic testing for OC risk and most of the men also said that they would support their female family members if they wanted to be tested. Several women said they would discuss this beforehand with their husbands and families and/or would share the result.

And then obviously going back and discussing it with family what’s come up as well.

FG3, woman, London.

Both men and women felt that some women would keep genetic testing and their result secret or ‘confidential’ from family and friends:

Some people may not want to share it with their family, want to keep it to themselves.

FG2, woman, London.

Some women who found the PROMISE programme personally acceptable, had concerns that younger women found to be at high risk for OC could have difficulties finding a husband due to the pro-natalist (promotion of human reproduction) nature of SA culture that favours the healthiest women. Others expressed concern that identifying someone as being at high risk or deciding to remove the ovaries could jeopardise existing marriages.

For peer review only - http://bmjopen.bmj.com/site/about/guidelines.xhtml

...if you get to our age then we would say yes. But for people like my daughter I would advise her not to. Because maybe the husband would leave the wife.

FG2, woman, London.

Some men indicated that they would not ask a potential partner about their cancer risk and that this would not influence their marital choice. Others spoke of the cultural importance of marriage and a woman's ability to bear children where removal of the ovaries would be a serious issue.

A few participants expressed the view that illnesses such as cancer are predestined and come from God. However, they did not suggest that this meant they would do nothing to prevent illness; instead religion was referred to as a coping resource.

...if I find out that I have got this problem; there will going to be ovarian cancer. I would thank God for giving me time to do what I want

FG1, woman, London.

5. Accessing services

Participants were eager for more information to be provided and for OC awareness campaigns within their communities.

They don't even know where it is...Where is the problem? How does it happen? It is very important that we give this information first.

FG4, woman, Luton.

A variety of methods to reach SA women with key OC prevention, earlier diagnosis and risk management messages were discussed including community based group sessions and campaigns in the local media targeted at those for whom English is not a first language. Several participants suggested that as English was not their first language or because they could not read English, they

For peer review only - http://bmjopen.bmj.com/site/about/guidelines.xhtml

would likely encounter difficulties in accessing genetic and screening services.

Both men and women indicated that, depending on the individual and situation, SA women would need or prefer to see a female healthcare professional, particularly if a physical examination was needed.

> *Mostly, the issues are about female doctors checking female things and male doctors checking males.*
>
> FG5, man, Luton.

Discussion

In this qualitative study, we identified a worrying lack of awareness of OC among SA women and men. Others have reported low awareness of cancer risk factors and symptoms amongst UK ethnic minority groups [23 24 25 26], although research has also shown a lack of ovarian cancer symptom awareness amongst the UK general population[26]. Irrespective of whether risk-stratified ovarian cancer management is offered to the public, improving awareness of OC amongst SAs is a health priority.

Some participants found it difficult to understand the brief presentation provided in the focus groups: it sometimes took several explanations to ensure that participants understood that genetic testing provides information on cancer risk rather than a diagnosis and that high risk did not mean that a person would definitely get cancer. As previously reported [11 12] participants tended to dichotomise risk as either high or low, with little discussion of intermediate risk. This underlines the need to develop optimal methods of conveying both the concept of OC risk and its meaning to the individual [26, 27]. The challenges of communicating risk estimates to the lay public are well documented and are particularly challenging [27-29] when information materials need to be acceptable to diverse populations.

For peer review only - http://bmjopen.bmj.com/site/about/guidelines.xhtml

Our main finding that attitudes toward genetic testing for OC risk and stratified management were mostly positive is consistent with other studies [30] but our study identified important cultural nuances. Participants maintained that personal genetic testing would not be viewed negatively from a religious standpoint and whilst a few referred to illness and death as predestined or from God, they indicated that it was still necessary to take action to maintain good health and, as in other research[31], religion was referred to as a coping strategy. Cancer fatalism was infrequently identified, but in this and other research [14], it was clear that the process of genetic testing and the receipt of a high risk result was anticipated by several participants to create heightened anxiety. Genetic testing for OC risk and stratified management may not be acceptable to all SA women, in particular younger women. Whilst the majority of women in this study indicated that they would accept a genetic test for OC risk if offered, many were already married and had children. Echoing research with UK Pakistanis about prenatal genetic testing [32 33] and UK SA women with breast cancer[34], some participants were concerned that illness or being identified as at high risk of OC could damage younger women's marriage prospects or cause marital problems. Participants acknowledged that not all SA women would discuss genetic testing or results with their family. Reluctance to discuss illness with family and friends due to taboo and perceived stigma was identified in the current study as well as in several other studies with SA participants [34 35], and could act as a barrier to the uptake of genetic testing.

Participants accepted the idea of stratified risk management, i.e. that there would be different management options for women with different levels of risk. With regard to the screening element of risk management, uptake of breast and cervical screening in the UK is lower amongst SA than White women[36]. Whilst the situation is slowly improving among SA women generally, change has not been significant for Muslim SA women [36]. Interestingly, in our study several groups discussed a positive cultural change in attitudes towards cancer screening and the majority reported attendance

For peer review only - http://bmjopen.bmj.com/site/about/guidelines.xhtml

at breast and/or cervical screening. In line with previous research [37-39], participants argued that lack of awareness, embarrassment and shyness were barriers to attending screening. Whilst OC screening with blood tests and ultrasound scans was seen as acceptable by most participants, the study did not explore the acceptability of *trans-vaginal* ultra-sound (the most commonly used scan to help detect OC), as this was beyond the scope of the study. However, as reported elsewhere [15 31], participants did have a preference for consultations with a gender-matched healthcare professional, particularly if a physical examination was needed.

Risk-reducing oophorectomy was seen as a particular dilemma, principally due to the importance placed on women's ability to bear children. Some male participants felt that it would be better to wait and see if a cancer develops, catch it at an early stage, and then have surgery. It may be that these men were inappropriately applying to OC their knowledge of how other cancers present and develop and this needs further investigation. However, apprehension about this surgery is not unique to SAs [40]. Our study highlights the need for sufficient information and support to be offered to SA women considering predictive genetic testing, and particularly for those with increased risk who will need to make risk management decisions.

This is the first study to explore UK SAs' perspectives on population-based genetic testing and risk stratified management for ovarian cancer, and includes participants with various levels of English language who are often not included in research. However, the opinions expressed by participants with regard to genetic testing and the PROMISE programme were based on brief information which was new to all, and related issues such as insurance and ethics were not spontaneously discussed. Furthermore, the current study did not inform patients that an increased risk of OC due to a *BRCA* gene mutation also indicates an increased risk of breast cancer, as this was beyond the scope of the study. The risk of breast cancer would further complicate decision-making as high risk patients would need to consider increased surveillance for breast cancer or risk-reducing mastectomy. Also,

For peer review only - http://bmjopen.bmj.com/site/about/guidelines.xhtml

both male focus groups were run by female facilitators; whilst participants did not express dissatisfaction with this, it may have influenced their responses.

Conclusions

Population-based risk assessment and stratified management may be acceptable to many SA men and women in the UK. Attitudes towards cancer screening were positive; however, opinions on risk-reducing surgery were mixed. The study highlights a need for tailored OC awareness campaigns within SA communities. To be inclusive, genetic testing and aftercare services should accommodate non-English speakers, offer appointments with a gender-matched healthcare professional, and offer patients support with their healthcare decisions.

For peer review only - http://bmjopen.bmj.com/site/about/guidelines.xhtml

Table 1. Sample demographics (n= 62)

	n (%)
**Gender**	
Female	49 (79.0)
Male	13 (21.0)
**Age**	
Mean years (range)	50.5 (22 - 82)
**Ethnic group**	
Bangladeshi	31 (50.0)
Indian	14 (22.6)
Pakistani	15 (24.2)
Other, Kashmiri	2 (3.2)
**Approx. years lived in the UK**	
Mean (range)	28.0 (2 - 49)
**First language***	
English	8 (12.9)
Bengali/Bangla	32 (51.6)
Gujarati	3 (4.8)
Hindi	5 (8.1)
Pahari	5 (8.1)
Punjabi	4 (6.5)
Sylheti	1 (1.6)
Urdu	15 (24.2)
Missing	1 (1.6)
**Religion**	
Hindu	7 (11.3)
Muslim	52 (83.9)
Sikh	3 (4.8)
**Marital status**	
Married/living with partner	44 (71.0)
Single/separated/divorced/widowed	17 (27.4)
Missing	1 (1.6)
**Employment**	
Full-time employment	4 (6.5)
Part-time employment	8 (12.9)
Homemaker	14 (22.6)
Retired	14 (22.6)

For peer review only

For peer review only - http://bmjopen.bmj.com/site/about/guidelines.xhtml

Disabled/too ill to work/ fulltime carer	3 (4.8)
Unemployed	19 (30.6)
**Education**	
Degree or higher	12 (19.4)
Qualification below degree level	19 (30.64)
Still studying	1 (1.8)
Other	11 (17.7)
No formal qualifications	19 (30.6)
**Attended screening**	
Amongst female participants (Breast or cervical screening or FOBT)	39 (79.6)
Amongst male participants (FOBT)	2 (15.4)
**Cancer within social network**	
Yes	29 (46.8)
No/Not sure/Prefer not to say	33 (53.2)
**Personal cancer diagnosis**	
Yes	3 (4.8)
No/Not sure/Prefer not to say	59 (95.2)

*Some participants had more than one first language.

For peer review only - http://bmjopen.bmj.com/site/about/guidelines.xhtml

Acknowledgements

This work was carried out at UCLH/UCL within the Cancer Theme of the NIHR UCLH/UCL Comprehensive Biomedical Research Centre supported by the UK Department of Health for the PROMISE study team.

We thank all the women and men who took part in the focus groups and all the centres and community group leaders who helped to recruit participants and distribute posters for the study.

Disclosure of interests

All authors declare that they have no competing interests.

Contribution to authorship

Authors KH, NA, LF, LS, JW, SS and AL contributed to the design of the study including refinement of the discussion guide. AL was overall responsible for the delivery of the project. NA and KH recruited participants and conducted the 7 focus group discussions. KH analysed the data and NA and AL checked the data for any divergent cases. Independent researcher SG performed coding checks on a proportion of the data. Authors KH, NA, LF, LS, JW, SS, SG, and AL contributed to interpretation of the results. KH drafted the manuscript and authors KH, NA, LF, LS, JW, SS, SG and AL critically reviewed and approved the manuscript.

Details of ethics approval

Approval to conduct the study was granted by the UCL Research Ethics Committee (project ID: 8053/003).

Funding

This work was supported by The Eve Appeal (509050) and Cancer Research UK (C1005/A12677). The

For peer review only - http://bmjopen.bmj.com/site/about/guidelines.xhtml

funders had no role in the study design; collection, management, analysis, or interpretation of data; writing of the report; or the decision to submit the report for publication.

Data sharing statement

Anonymised qualitative data is available on request.

Supplementary files

COREQ checklist

Focus Group Discussion Guide (Women)

Focus group Discussion Guide (Men)

Focus Group Information slides

For peer review only - http://bmjopen.bmj.com/site/about/guidelines.xhtml

References

1. Cancer Research UK. Ovarian cancer incidence by UK country. Secondary Ovarian cancer incidence by UK country 2016. http://www.cancerresearchuk.org/health-professional/cancer-statistics/statistics-by-cancer-type/ovarian-cancer/incidence#heading-Zero.
2. Bannister N, Broggio J. Cancer survival by stage at diagnosis for England (experimental statistics): Adults diagnosed 2012, 2013 and 2014 and followed up to 2015., 2016.
3. Ovacome. Secondary. http://www.ovacome.org.uk/information/screening/.
4. Menon U, Gentry-Maharaj A, Hallett R, et al. Sensitivity and specificity of multimodal and ultrasound screening for ovarian cancer, and stage distribution of detected cancers: results of the prevalence screen of the UK Collaborative Trial of Ovarian Cancer Screening (UKCTOCS). *The Lancet Oncology* 2009;10:327-40.
5. Rosenthal AN, Fraser LS, Philpott S, et al. Evidence of stage shift in women diagnosed with ovarian cancer during phase II of the United Kingdom Familial Ovarian Cancer Screening Study. *Journal of Clinical Oncology* 2017:JCO-2016.
6. Jacobs I, Menon U, Ryan A, et al. Ovarian cancer screening and mortality in the UK Collaborative Trial of Ovarian Cancer Screening (UKCTOCS): a randomised controlled trial. *The Lancet* 2016;387(10022):945-56.
7. Antoniou A, Pharoah PD, Narod S, et al. Average risks of breast and ovarian cancer associated with BRCA1 or BRCA2 mutations detected in case series unselected for family history: a combined analysis of 22 studies. *The American Journal of Human Genetics* 2003;72(5):1117-30.
8. Manchanda R, Jacobs I. Genetic screening for gynecological cancer: where are we heading? *Future Oncology* 2015;12(2):207–20.
9. Burton H, Chowdhury S, Dent T, et al. Public health implications from COGS and potential for risk stratification and screening. *Nature Genetics* 2013;45(4):349.
10. Davies SC. Annual report of the Chief Medical Officer 2016, Generation genome. In: Health Do, ed. London, 2017.
11. Meisel SF, Side L, Fraser L, et al. Population-based, risk-stratified genetic testing for ovarian cancer risk: A focus group study. *Public Health Genomics* 2013;16:184-91.
12. Rahman B, Meisel SF, Fraser L, et al. Population-based genetic risk prediction and stratification for ovarian cancer: views from women at high risk. *Familial Cancer* 2015;14(1):135-44.
13. The Office for National Sataistics. Ethnicity and national identity in England and Wales: 2011. Secondary Ethnicity and national identity in England and Wales: 2011 2011. http://www.ons.gov.uk/peoplepopulationandcommunity/culturalidentity/ethnicity/articles/ethnicityandnationalidentityinenglandandwales/2012-12-11.
14. Hann KEJ, Freeman M, Fraser L, et al. Awareness, knowledge, perceptions, and attitudes towards genetic testing for cancer risk among ethnic minority groups: a systematic review. *BMC Public Health* 2017;17(1):503.
15. Atkin K, Ali N, Chu CE. The politics of difference? Providing a cancer genetics service in a culturally and linguistically diverse society. *Diversity in Health and Care* 2009;6(3):149–57.
16. Genetic Alliance UK. Identifying family risk of cancer: Why is this more difficult for ethnic minority communities and what would help? London, 2012.
17. Burr V. *An introduction to social constructionism*. London: Routledge, 1995.
18. Kitzinger J. Qualitative research. Introducing focus groups. *BMJ* 1995;311:299-302.
19. Morse JM. Determining sample size. *Qualitative Health Research* 2000;10(1):3-5.
20. Huo D, Olopade OI. Genetic testing in diverse populations: are researchers doing enough to get out the correct message? . *The Journal of the American Medical Association* 2007;298(24):2910-11.
21. Braun V, Clarke V. Using thematic analysis in psychology. *Qualitative Research in Psychology* 2006;3(3):77-101.

For peer review only - http://bmjopen.bmj.com/site/about/guidelines.xhtml

22. Tong A, Sainsbury P, Craig J. Consolidated criteria for reporting qualitative research (COREQ): a 32-item checklist for interviews and focus groups. *Int J Qual Health Care* 2007;19(6):349-57.
23. Waller J, Robb K, Stubbings S, et al. Awareness of cancer symptoms and anticipated help seeking among ethnic minority groups in England. *British Journal of Cancer* 2009;101:S24-S30.
24. Marlow LAV, Robb KA, Simon AE, et al. Awareness of cancer risk factors among ethnic minority groups in England. *Public Health* 2012;126(8):702-09.
25. Fallowfield L, Fleissig A, Barrett J, et al. Awareness of ovarian cancer risk factors, beliefs and attitudes towards screening: baseline survey of 21 715 women participating in the UK Collaborative Trial of Ovarian Cancer Screening. . *British Journal of Cancer* 2010;103(4):454-61.
26. Low EL, Waller J, Menon U, et al. Ovarian cancer symptom awareness and anticipated time to help-seeking for symptoms among UK women. *Journal of Family Planning and Reproductive Health Care* 2013;39:163-71.
27. Janssen E, van Osch L, de Vries H, et al. The influence of narrative risk communication on feelings of cancer risk. . *British Journal of Health Psychology* 2013;18(2):407-19.
28. Han PKJ, Klein WMP, Lehman T, et al. Communication of uncertainty regarding individualized cancer risk estimates: Effects and influential factors. *Medical Decision Making* 2011;31:354-66.
29. Peters E, McCaul KD, Stefanek M, et al. A heuristics approach to understanding cancer risk perception: Contributions from judgment and decision-making research. *Annals of Behavioral Medicine* 2006;31(1):45-52.
30. Lerman C, Daly M, Masny A, et al. Attitudes about genetic testing for breast-ovariancancer susceptibility. *Journal of Clinical Oncology* 1994;12(4):843-50.
31. Thomas VN, Saleem T, Abraham R. Barriers to effective uptake of cancer screening among black and minority ethnic groups. *International Journal of Palliative Nursing* 2005;11(11):562-71.
32. Shaw A, Hurst JA. 'I don't see any point in telling them': attitudes to sharing genetic information in the family and carrier testing of relatives among British Pakistani adults referred to a genetics clinic. *Ethnicity & Health* 2009;14(2):205-24.
33. Darr A, Small N, Ahmad WI, et al. Examining the family-centred approach to genetic testing and counselling among UK Pakistanis: a community perspective. *Journal of Community Genetics* 2013;4(1):49-57.
34. Karbani G, Lim JNW, Hewison J, et al. Culture, attitude and knowledge about breast cancer and preventive measures: a qualitative study of South Asian breast cancer patients in the UK. *Asian Pacific Journal of Cancer Prevention* 2011;12(6):1619-26.
35. Randhawa G, Owens A. The meanings of cancer and perceptions of cancer services among South Asians in Luton. *British Journal of Cancer* 2004;91(1):62-68.
36. Szczepura A, Price C, Gumber A. Breast and bowel cancer screening uptake patterns over 15 years for UK south Asian ethnic minority populations, corrected for differences in socio-demographic characteristics. *BMC Public Health* 2008;8(1):346.
37. Marlow LAV, Waller J, Wardle J. Barriers to cervical cancer screening among ethnic minority women: a qualitative study. *Journal of Family Planning and Reproductive Health Care* 2015;0:1-7.
38. Crawford J, Ahmad F, Beaton D, et al. Cancer screening behaviours among South Asian immigrants in the UK, US and Canada: a scoping study. *Health and Social Care in the Community* 2016;24(2):123–53.
39. Robb K, Wardle J, Stubbings S, et al. Ethnic disparities in knowledge of cancer screening programmes in the UK. *Journal of Medical Screening* 2010;17(3):125-31.
40. Tong A, Kelly S, Nusbaum R, et al. Intentions for risk-reducing surgery among high-risk women referred for BRCA1/BRCA2 genetic counseling. *Psycho-Oncology* 2015;24:33–39.

For peer review only - http://bmjopen.bmj.com/site/about/guidelines.xhtml

Title: Attitudes towards a programme of risk assessment and stratified management for ovarian cancer: A focus group study of UK South Asians' perspectives

Focus Group Discussion Guide (Women)

1. Introduction

- Welcome and thanks for taking part
- Introduction and purpose of the study
- Confidentiality (agree that the discussion should be confidential among respondents)
- Timing (up to 1 ½ hours)
- Reminder of audio-recording
- Anonymity in report writing etc.
- Ground rules – respect different opinions, keep mobile phones off or on silent.
- No right/wrong answers – hoping for a range of views
- Participants introduce themselves

2. General awareness/attitudes towards ovarian cancer risk

I would like to start by discussing what you know about ovarian cancer and what you think about your ovarian cancer risk

- What do you know about ovarian cancer?
  - Prompts: What do you know about how common ovarian cancer is/symptoms/ causes/ risk factors/reducing risk?
- What do you think about your own risk of ovarian cancer?
  - Prompt: Do you think your chances of developing ovarian cancer are the same as the rest of the UK population or higher or lower?

***Focus group participants presented with slides on genetic risk.**

3. Opinions on genetic information

We are working on a project where we will be inviting women to have genetic testing for ovarian cancer risk, regardless of whether they have a family history of cancer.

- Does genetic cancer risk information make sense to you?
  - What did you know about genetic testing for cancer risk before coming to this discussion today?
  - Does the link between genetics and risk of ovarian cancer make sense?
- How would you feel about having genetic testing and receiving test results on ovarian cancer risk?
  - Prompt: Pros/cons of testing?
  - Prompt: Pros/cons of finding out test result?
- What might influence your decision to have genetic testing for ovarian cancer risk if it were offered?
  - Prompt: Friends/ Family/ Religion/ Culture/ Access/ Practical issues/ Concerns?
- How do you think your family and friends would view your decision of having (or not having) genetic testing for risk of ovarian cancer?
  - Prompt: Husband/ Sisters/ Parents/ Children/ Close female friends?

For peer review only

4. Opinions of risk stratification approach and possible risk management options

In our project, women will be told whether they have a low, intermediate or high risk for ovarian cancer, based on a combination of their genetic risk and other risk factors. Identifying other risk factors is done using questions about family history, lifestyle and health information. We expect that most of the women will have a low risk result, fewer will have an intermediate risk, and fewer still will have a high risk. Being at low risk would not mean that the women have no risk of developing ovarian cancer.

Depending on their risk level, women would be offered different interventions. Women at low risk would receive information telling them that they are low risk and don't need further monitoring, they would also be given information about symptoms of ovarian cancer to be aware of. Women at intermediate risk would be offered screening every four months involving a blood test and a yearly ultrasound scan. Preventative surgery, involving the removal of the ovaries, would be discussed as an option depending on the characteristics of the woman (such as age, their family history of cancer etc). For high risk women, preventative surgery would be offered as a main option, but screening would also be discussed with these patients.

We are wondering whether women might be interested in taking part in this research and how women might feel about it if it were broadened out into a general population service.

- What do you think about this idea?
  - Prompt: Pros and cons?
  - Prompt: Culturally acceptable? Religiously acceptable?
  - Prompt: What do you think about the idea of offering different care to women depending on their level of risk?
  - Prompt: What do you think about the risk management options?
- How would you feel if you were in the low/intermediate/high risk management group?
  - Prompt: Pros and cons?
  - Prompt: How might it impact you/ your family?
- How would you feel about taking part in this project if you were invited?
  - Prompt: Would you be interested in taking part?

Much of what we know about ovarian cancer risk due to inherited genetics comes from research with white women of European descent. Whilst there is no evidence to suggest that this risk differs between ethnic groups, we don't know for certain. It is possible that the estimated cancer risk based on previous research may be less accurate for other ethnic groups.

- How might this information impact on whether or not you would agree to have your cancer risk estimated from genetic and other information/ impact on taking part in the trial if invited?

- How might this programme be provided in a way that you would find acceptable and accessible?
  - Prompt: What would be important to you about how the service is provided?
  - Prompt: Barriers/facilitators?

5. Final comments

- Is there anything else that you'd like to talk about that you think might be relevant?

6. Debrief and thank participants

For peer review only

Title: Attitudes towards a programme of risk assessment and stratified management for ovarian cancer: A focus group study of UK South Asians' perspectives

Focus Group Discussion Guide (Men)

1. Introduction

- Welcome and thanks for taking part
- Introduction and purpose of the study
- Confidentiality (agree that the discussion should be confidential among respondents)
- Timing (up to 1 ½ hours)
- Reminder of audio-recording
- Anonymity in report writing etc.
- Ground rules – respect different opinions, keep mobile phones off or on silent
- No right/wrong answers – hoping for a range of views
- Participants introduce themselves

2. General awareness/attitudes towards ovarian cancer risk

- I'd like to start by discussing what you know about ovarian cancer?
    - Prompts: What do you know about how common ovarian cancer is/symptoms/ causes/ risk factors/reducing risk?

***Focus group participants presented with slides on genetic risk.**

3. Opinions on genetic information

We are working on a project where we will be inviting women to have genetic testing for ovarian cancer risk, regardless of whether they have a family history of cancer.

- Does genetic cancer risk information make sense to you?
    - What did you know about genetic testing for cancer risk before coming to this discussion today?
    - Does the link between genetics and risk of ovarian cancer make sense?
- How would you feel about female members of your family (your wife/ sister/ mother/ daughter) having genetic testing and receiving test results on ovarian cancer risk?
    - Prompt: pros/cons? Culturally acceptable? Religiously acceptable?
    - Prompt: What impact could it have on you/your family?

4. Opinions of risk stratification approach and possible risk management options

In our project, women will be told whether they have a low, intermediate or high risk for ovarian cancer, based on a combination of their genetic risk and other risk factors. Identifying other risk factors is done using questions about family history, lifestyle and health information. We expect that most of the women will have a low risk result, fewer will have an intermediate risk, and fewer still will have a high risk. Being at low risk would not mean that the women have no risk of developing ovarian cancer.

Depending on their risk level, women would be offered different interventions. Women at low risk would receive information telling them that they are low risk and don't need further monitoring, they would also be given information about symptoms of ovarian cancer to be aware of. Women at intermediate risk would be offered screening every four months involving a blood test to check for levels of a biomarker and a yearly ultrasound scan. Preventative surgery, involving the removal of the

For peer review only

ovaries, would be discussed as an option depending on the characteristics of the woman (such as age, their family history of cancer etc). For high risk women, preventative surgery would be offered as a primary option, but screening would also be discussed with these patients.

- What do you think about this idea?
  - Prompt: Pros and cons?
  - Prompt: Culturally acceptable? Religiously acceptable?
  - Prompt: What do you think about the idea of offering different care/options to women depending on their level of risk?
  - Prompt: What do you think about the risk management options (information/screening/surgery)?
  - Thinking about wife/mother/sister: what impact do you think a high/ intermediate/ low risk would have for them/the family?

Much of what we know about ovarian cancer risk due to inherited genetics comes from research with white women of European descent. Whilst there is no evidence to suggest that this risk differs between ethnic groups, we don't know for certain. It is possible that the estimated cancer risk based on previous research may be less accurate for other ethnic groups.

- How might this information impact on whether or not you would support or encourage female family members' decision to have their cancer risk estimated from genetic and other information?

- How might this programme of genetic testing and risk management be provided in a way that would be acceptable and accessible?
  - What would be important about how the service is provided?
  - Barriers/facilitators?

<u>5. Final comments</u>

- Is there anything else that you'd like to talk about that you think might be relevant?

<u>6. Debrief and thank participants</u>

For peer review only

UCL

Ovarian cancer risk

- The lifetime risk of a women in the UK getting ovarian cancer is about 2%.
- This means around 1 in 50 women will develop ovarian cancer at some point in their life.

For peer review only - http://bmjopen.bmj.com/site/about/guidelines.xhtml

Ovarian cancer risk

- We don’t know the cause of most ovarian cancers.
- We know some of the ‘**risk factors’ –** these are things that may increase the chances of developing cancer.
- Having a cancer risk factor doesn’t mean that a person will definitely get cancer – just as not having it doesn’t mean that they won’t.

UCL

Genes

- Genes carry the biological information passed from parent to child.
- Some genes are known to increase the risk of ovarian cancer.
- Changes (mutations) in certain genes are known to increase the risk of cancer.

Ovarian cancer genes

- Mutations in two genes – called BRCA1 and BRCA2 – increase the risk of ovarian cancer.
- BRCA1 and BRCA2 mutations are very rare but family members who inherit them have a much greater risk of ovarian cancer.
- Scientists can also identify other relevant gene mutations involved.

Testing for genetic risk

- It is possible to test for BRCA1 and BRCA2 gene mutations.
- Genetic testing involves a blood test.
- Genetic material (DNA) is taken from the blood cells to test for mutations.
- At present genetic testing for ovarian cancer is not available on the NHS except for women with a strong family history of cancer.

For peer review only - http://bmjopen.bmj.com/site/about/guidelines.xhtml

Importance of family history of cancer

- Families with a strong ‘family history’ of ovarian cancer are more likely to carry the gene mutations.
- A strong family history means:
  - – Two close relatives (mother, sister, daughter) with ovarian cancer
  - – One close relative with ovarian cancer, and, on the same side of the family:
    - One close relative who had breast cancer before age 50
    - Two close relatives who had breast cancer before age 60
    - Three close relatives who had bowel or womb (uterus) cancer

UCL

PROMISE research programme

- Genetic testing + personal and lifestyle information = risk
- Women who agree to this will be grouped as being at **high**, **intermediate** or **low** risk for ovarian cancer.

PROMISE continued…

- The doctor would then discuss different risk management options:

➢ Low risk: symptom awareness information.

➢ Intermediate risk: screening, or surgery depending on age.

➢ High risk: surgery, or screening if not ready to have surgery.

COREQ (COnsolidated criteria for REporting Qualitative research) Checklist

A checklist of items that should be included in reports of qualitative research. You must report the page number in your manuscript where you consider each of the items listed in this checklist. If you have not included this information, either revise your manuscript accordingly before submitting or note N/A.

Topic	Item No.	Guide Questions/Description	Reported on Page No.
**Domain 1: Research team and reflexivity**			
Personal characteristics			
Interviewer/facilitator	1	Which author/s conducted the interview or focus group?	
Credentials	2	What were the researcher's credentials? E.g. PhD, MD	
Occupation	3	What was their occupation at the time of the study?	
Gender	4	Was the researcher male or female?	
Experience and training	5	What experience or training did the researcher have?	
Relationship with participants			
Relationship established	6	Was a relationship established prior to study commencement?	
Participant knowledge of the interviewer	7	What did the participants know about the researcher? e.g. personal goals, reasons for doing the research	
Interviewer characteristics	8	What characteristics were reported about the inter viewer/facilitator? e.g. Bias, assumptions, reasons and interests in the research topic	
**Domain 2: Study design**			
Theoretical framework			
Methodological orientation and Theory	9	What methodological orientation was stated to underpin the study? e.g. grounded theory, discourse analysis, ethnography, phenomenology, content analysis	
Participant selection			
Sampling	10	How were participants selected? e.g. purposive, convenience, consecutive, snowball	
Method of approach	11	How were participants approached? e.g. face-to-face, telephone, mail, email	
Sample size	12	How many participants were in the study?	
Non-participation	13	How many people refused to participate or dropped out? Reasons?	
Setting			
Setting of data collection	14	Where was the data collected? e.g. home, clinic, workplace	
Presence of non-participants	15	Was anyone else present besides the participants and researchers?	
Description of sample	16	What are the important characteristics of the sample? e.g. demographic data, date	
Data collection			
Interview guide	17	Were questions, prompts, guides provided by the authors? Was it pilot tested?	
Repeat interviews	18	Were repeat inter views carried out? If yes, how many?	
Audio/visual recording	19	Did the research use audio or visual recording to collect the data?	
Field notes	20	Were field notes made during and/or after the inter view or focus group?	
Duration	21	What was the duration of the inter views or focus group?	
Data saturation	22	Was data saturation discussed?	
Transcripts returned	23	Were transcripts returned to participants for comment and/or	

For peer review only

Topic	Item No.	Guide Questions/Description	Reported on Page No.
		correction?	
**Domain 3: analysis and findings**			
Data analysis			
Number of data coders	24	How many data coders coded the data?	
Description of the coding tree	25	Did authors provide a description of the coding tree?	
Derivation of themes	26	Were themes identified in advance or derived from the data?	
Software	27	What software, if applicable, was used to manage the data?	
Participant checking	28	Did participants provide feedback on the findings?	
Reporting			
Quotations presented	29	Were participant quotations presented to illustrate the themes/findings? Was each quotation identified? e.g. participant number	
Data and findings consistent	30	Was there consistency between the data presented and the findings?	
Clarity of major themes	31	Were major themes clearly presented in the findings?	
Clarity of minor themes	32	Is there a description of diverse cases or discussion of minor themes?	

Developed from: Tong A, Sainsbury P, Craig J. Consolidated criteria for reporting qualitative research (COREQ): a 32-item checklist for interviews and focus groups. *International Journal for Quality in Health Care*. 2007. Volume 19, Number 6: pp. 349 – 357

Once you have completed this checklist, please save a copy and upload it as part of your submission. DO NOT include this checklist as part of the main manuscript document. It must be uploaded as a separate file.

For peer review only

BMJ Open

Attitudes towards a programme of risk assessment and stratified management for ovarian cancer: A focus group study of UK South Asians' perspectives

Journal:	*BMJ Open*
Manuscript ID	bmjopen-2018-021782.R1
Article Type:	Research
Date Submitted by the Author:	09-May-2018
Complete List of Authors:	Hann, Katie; University College London; Royal Holloway, University of London, Health Psychology Research Unit<br>Ali, Nasreen; University of Bedfordshire Faculty of Health and Social Sciences, Institute for Health Research<br>Gessler, Sue; University College London Hospitals NHS Foundation Trust, ; UCL Institute for Women's Health and NIHR University College London Hospitals Biomedical Research Centre, Department of Women's Cancer<br>Fraser, Lindsay; Institute for Women's Health, Department of Women's Cancer<br>Side, Lucy; University College London; University Hospital Southampton<br>Waller, Jo; UCL, Epidemiology and Public Health<br>Sanderson, Saskia; University College London<br>Lanceley, Anne; UCL, Department of Women's Cancer
**Primary Subject Heading**:	Health services research
Secondary Subject Heading:	Genetics and genomics, Obstetrics and gynaecology, Oncology
Keywords:	ovarian cancer, genetic testing, risk assessment, risk stratification, Risk management < HEALTH SERVICES ADMINISTRATION & MANAGEMENT

SCHOLARONE™
Manuscripts

Title: Attitudes towards a programme of risk assessment and stratified management for ovarian cancer: A focus group study of UK South Asians' perspectives

Running title: Attitudes to ovarian cancer risk stratification.

Hann, K. E J.,[1,2] Ali, N.,[3] Gessler, S.,[1] Fraser, L.,[1] Side, L.,[1,4] Waller, J.,[5] Sanderson, S. C.,[5,6] & Lanceley, A.[1] for the PROMISE study team.

[1]Dept. of Women's Cancer, EGA UCL Institute for Women's Health, University College London, London, UK, WC1E 6AU.

[2]Health Psychology Research Unit, Royal Holloway, University of London, Egham, Surrey, UK, TW20 0EX.

[3]Institute for Health Research, University of Bedfordshire, Luton, Bedfordshire, UK, LU2 8LE.

[4]Clinical Genetics, University Hospital Southampton NHS Foundation Trust, Southampton, UK, SO16 5YA.

[5]Dept. Behavioural Science and Health, Institute of Epidemiology and Health Care, University College London, London, UK, WC1E 6BT.

[6] Great Ormond Street Hospital, London, UK, WC1N 3JH.

Corresponding Author:

Anne Lanceley, PhD

237c

Medical School Building

74 Huntley Street

London, WC1E 6AU

Email: a.lanceley@ucl.ac.uk

Phone: +4420 7679 6807

For peer review only - http://bmjopen.bmj.com/site/about/guidelines.xhtml

Abstract

Objective: Population-based risk assessment, using genetic testing, and the provision of appropriate risk management could lead to prevention, early detection and improved clinical management of ovarian cancer (OC). Previous research with mostly white British participants found positive attitudes towards such a programme, the current study aimed to explore the attitudes of South Asian (SA) women and men in the UK with the aim of identifying how best to implement such a programme to minimise distress and maximise uptake.

Design: Semi-structured qualitative focus group discussions.

Setting: Community centres across North London and Luton.

Participants: 49 women and 13 men who identified as SA (Indian, Pakistani or Bangladeshi), which constitutes the largest non-European ethnic minority group in the UK.

Methods: 7 community based focus groups were held. Group discussions were transcribed verbatim, coded and analysed thematically.

Results: Awareness and knowledge of OC symptoms and specific risk factors was low. The programme was acceptable to most participants and attitudes to it were generally positive. Participants' main concerns related to receiving a high risk result following the genetic test. Younger women may be more cautious of genetic testing, screening, or risk-reducing surgery due to the importance of marriage and child bearing in their SA cultures.

Conclusions: A crucial first step to enable implementation of population based genetic risk assessment and management in OC is to raise awareness of OC within SA communities. It will be important to engage with the SA community early on in programme implementation to address their specific concerns and to ensure culturally tailored decision support.

For peer review only - http://bmjopen.bmj.com/site/about/guidelines.xhtml

Strengths and limitations of this study

- This is the first study to explore the attitudes of a UK ethnic minority group towards population-based risk assessment and stratified management for ovarian cancer.
- The study explored the attitudes of both women and men.
- Opinions solicited during the focus groups were directly related to information provided about population-based risk assessment and stratified management for ovarian cancer and this may have limited responses.
- Two female researchers facilitated all the focus groups including those with men, this may have influenced the findings.

Key words: Ovarian cancer, genetic testing, risk assessment, risk stratification, risk management, South Asian

For peer review only - http://bmjopen.bmj.com/site/about/guidelines.xhtml

For peer review only

Introduction

Ovarian cancer (OC) is the sixth most common cancer amongst UK women[1]. Due to the non-specific symptoms associated with this cancer, diagnosis is usually at a late stage when prognosis is poor[2]. Earlier detection of OC could help to save lives and this has fuelled voluntary sector demands for research to investigate approaches for prevention and earlier diagnosis [3]. A definitive ongoing trial investigating screening for OC in postmenopausal women has shown this to be sensitive and feasible[4 5], but to date without a significant mortality benefit[5,6]. Nevertheless, a stage shift at diagnosis has been evidenced and for high risk women who are not ready to have risk-reducing surgery [5], screening could be an interim option.

Mutations of *BRCA1* and *BRCA2* genes considerably increase an individual's risk of OC;[7] combined with non-genetic information (e.g. family history of cancer, age, and lifestyle factors), this genetic information can be used to estimate a woman's risk. Following risk assessment, risk stratified management could benefit patients by identifying those at high risk and in most need of management, whilst avoiding over-investigation of those at lowest risk[8 9]. A current programme of research, Predicting Risk of Ovarian Malignancies, Improved Screening and Early Detection (PROMISE, https://eveappeal.org.uk/our-research/our-research-programmes/promise-2016/), involves a feasibility trial to investigate whether stratified OC risk management is acceptable to women in the UK general population. In this programme, women will be provided with an estimate of their OC risk and stratified as low, intermediate or high risk. Those with the lowest risk will be provided with information on OC. Screening, or surgery to remove the ovaries, will be offered to those at intermediate and high risk. In the future, equivalent programmes could be rolled out for other cancers[10].

Positive attitudes to the PROMISE programme were reported amongst women in the general [11] and in high OC risk populations[12], but study samples did not reflect the diverse UK population. A key

For peer review only - http://bmjopen.bmj.com/site/about/guidelines.xhtml

concern for any public health programme is its inclusivity, yet little is known about UK ethnic minority peoples' awareness and attitudes toward genetic testing for cancer risk [13]. The few studies that have explored delayed use of genetic services among UK minority groups identify low awareness of their availability, language barriers, and unwillingness to discuss cancer due to stigma and fear as contributing factors[14 15].

This study aimed to explore South Asian (SA) women's and men's attitudes towards the PROMISE programme and the idea of population-based genetic testing and risk stratified management of OC, and to identify factors which may influence participation. The SA community is the largest non-European ethnic minority group in the UK, over 5% of the population in England and Wales identify as SA (Pakistani, Bangladeshi and Indian)[16]. The attitudes of both men and women were explored because it is known that healthcare decisions may be influenced by family members including husbands and/or fathers[17 18].

Methods

Methodological approach

The study took a constructionist perspective in which meaning and experience are considered to be socially produced and reproduced rather than as immutable individual characteristics[19]. It used a qualitative research design of focus groups to explore existing knowledge of OC and views of the novel population-based risk management intervention. Focus groups are well suited to exploration of public health topics and are a good way of identifying community norms and cultural values[20]. Structured discussion within the groups provided an opportunity for participants to question each other and reflect upon and challenge one another's views. Thematic analysis of these data was undertaken.

Patient/public involvement

For peer review only - http://bmjopen.bmj.com/site/about/guidelines.xhtml

Patients and the public were involved as project steering group members in the design of the overall PROMISE programme and the health behaviour work-stream within which this study was delivered. Members of the SA community also contributed by pilot testing our presentation materials for the focus groups.

Setting

Participants were recruited from the North London Boroughs of Brent, Newham and Tower Hamlets and Luton areas which have large SA settler communities. Groups were conducted in suitable local community venues between November 2016 and April 2017. Approval to conduct the study was granted by the UCL Research Ethics Committee (project ID: 8053/003).

Participants

Purposive sampling was used to include only individuals ≥18 years old, who self-identified as being of South Asian ethnicity (Indian, Pakistani, Bangladeshi) and to include a wide spread of ages. At least some conversational English language was needed to take part. Men were included in the study as they may play a role in supporting and advising female family members' healthcare decisions. Women were excluded if they had (a) a diagnosis of OC and/or (b) previously had genetic testing to find out about personal cancer risk.

SA women and men were introduced to the study by local community centre staff (N= 53), and through poster and leaflet advertisement at community centres and by a local women's health organisation (N= 9). The few eligible individuals who contacted the research team directly by phone or email were sent the study information and had an opportunity to ask questions. We aimed to obtain a broad range of views and continued to recruit until we achieved data saturation,[21] when no new views were being expressed. Of those who agreed to participate, 2 withdrew due to sickness. Participants received a £20 gift voucher and travel costs.

For peer review only - http://bmjopen.bmj.com/site/about/guidelines.xhtml

Data collection

Seven focus group discussions were held at community centres: five with women (n=12, n=8, n=9, n=11, n=9) and two with men (n=7, n=6). Each discussion lasted approximately 75 minutes. Groups were facilitated by NA, a SA multilingual senior qualitative researcher, and KH, a research assistant with a Masters level qualification, acting as moderator and note-taker alternatively. A semi-structured discussion guide developed from previous work [11] and the literature and which comprised open-ended, none-directive questions, was used (See Additional Files 1 & 2). These aimed to facilitate discussion and elicit participant views.

At the start of each focus group KH and NA introduced themselves briefly (name, job, associated university), stated the study's purpose and confirmed what participation involved. Intra-group confidentiality, audio–recording and study report confidentiality were highlighted with an opportunity to ask questions. Participants gave written consent and each completed a demographic questionnaire.

To open the discussion participants were invited to share their current awareness and knowledge of OC. Essential information concerning OC, including the increased risk amongst those with *BRCA1/2* gene mutations, the possibility to test for these and the PROMISE programme's proposal to offer OC risk-stratified management (see Additional file 3), was then given in a short slide presentation and hand-out. It was also explained that OC risk information from genetic testing could be less accurate for women of SA ethnicity as most research has been carried out with women of European descent[22]. The presentation text was designed to be understood by participants irrespective of educational attainment and was pilot tested with SA women for comprehension.

Two groups were conducted solely in English, and five in multiple languages including English, Urdu, Hindi, Punjabi, Pahari and Bengali. In two groups involving Bangladeshi women, those fluent in

For peer review only - http://bmjopen.bmj.com/site/about/guidelines.xhtml

English assisted their peers so that everyone understood the language used. In one group which included Bangladeshi men a woman acted as a translator to help a few participants take part in the discussion.

Analysis

Group discussions were audio recorded, translated into English if necessary, transcribed verbatim by a professional multi-lingual transcription service, and checked against the recordings for accuracy by KH and NA. The data was analysed thematically [23] using QSR International's NVivo 10 Software (2012). KH read and re-read the transcripts and generated initial codes. AL and NA also read the transcripts to identify any divergent cases, and initial codes were refined after discussion. Themes were identified deductively, guided by the discussion topics, and inductively, as they emerged from the data. KH analysed all seven transcripts and an independent researcher (SG) coded two transcripts. KH and SG met to confirm any divergent cases and discuss any disagreements in coding until a consensus was reached. This paper follows the consolidated criteria for reporting qualitative studies (COREQ) [24].

Findings

A total of 49 women and 13 men took part. Demographic characteristics of participants are presented in Table 1. Five themes were identified: participants' awareness and knowledge of OC and genetic risk; attitudes towards genetic testing and finding out about OC risk; attitudes towards risk stratified management; family, culture and religion; and accessing services.

1. Awareness and knowledge of OC and genetic risk

The term ovary/ovaries was not familiar to many participants. In all groups there was some confusion over the ovaries, what they are and where they are located in the body. Some participants had difficulty distinguishing between the ovaries and the womb and this was reflected in some Urdu

For peer review only - http://bmjopen.bmj.com/site/about/guidelines.xhtml

speakers using the word 'bacha daani' (womb) and 'undah daani' (ovaries) interchangeably. Most participants were aware of the UK's common cancers mentioning breast, prostate, and lung, as well as cervical cancer, and correctly named some of the main risk factors for these. However, the majority had not come across OC and were unaware of the risk factors and main symptoms of the disease.

Ovarian cancer.....no one's heard of it

FG4, woman, Luton.

…I'm quite aware, I do pick up leaflets and read but I don't think I've come across ovarian cancer, not on TV, not on any sort of media, not on the train, nothing.

FG3, woman, London.

A few women incorrectly believed that use of hormone replacement therapy or the contraceptive pill would increase a woman's risk of OC. Older age was infrequently reported as a risk factor and few participants spontaneously spoke about family history or genetic risk. A minority of participants who demonstrated awareness of OC explained that this was due to either having researched the topic online prior to the group discussion (n=1) or from experience of a relative with OC (n=1).

Likely due to the lack of awareness, the women had not considered their risk of OC. Some indicated that they did not generally think about their personal risk of cancer. When asked, most acknowledged that they would have some risk of OC, although whether they perceived this to be the same, lower, or higher than others in the general population varied within and between the groups.

Other populations, I think it's the same?

FG1, woman, London.

It's higher in Asian

For peer review only - http://bmjopen.bmj.com/site/about/guidelines.xhtml

But tell me this, I have never heard of any Asian person with ovarian cancer

FG4, woman, Luton.

Most participants had not heard of genetic testing for cancer risk and those that had did not know about the specific *BRCA1/2* genes. After participants had been informed about genetic testing within the group it became apparent that some had difficulty understanding that a) the test would provide information about a person's risk rather than a cancer diagnosis, and b) being at risk did not mean that they would definitely develop cancer.

2. Attitudes toward genetic testing and finding out about OC risk

Based on our presentation of genetic risk and genetic testing for OC, in discussion most participants initially expressed positive views. They felt they would benefit from knowing if they were at increased risk because they could take steps to manage their individual risk.

So you are aware of it, and you know how to prevent it, getting information, what are the risks, and how to do your daily activity, your daily lifestyle, maybe that can change …

FG2, woman, London.

The majority of women indicated that they would accept genetic testing, and several men said that they would encourage female family members to have testing if it were offered, although they acknowledged that ultimately it would be the individual's decision. Many participants remained positive about genetic testing even after being told that risk information could potentially be less accurate for ethnic minorities. Participants said there were no cultural or religious prohibitions on genetic testing for cancer risk and these aspects of the discussion prompted some Muslim participants to speak of the positive influence that religion has on maintaining good health. In one group, participants referred specifically to the Imam (Muslim religious scholar), and his role in providing guidance to the community on health practices.

For peer review only - http://bmjopen.bmj.com/site/about/guidelines.xhtml

Your religion wants you to look after yourself.

FG1, woman, London.

The main concerns voiced about genetic testing for OC risk related to experiencing worry between the blood test and receiving the result, and fear regarding the psychological impact of a high risk result and what such a result would lead to if received:

"Until you know the outcome, your brain will be working overtime"

FG4, woman, Luton.

Participants were not fearful of providing blood for the genetic test and some indicated that whilst they were positive about genetic testing others might be wary since they may be unfamiliar with OC, genetic testing, and fearful of cancer per se.

"Negative side could be some people, maybe my mother, wouldn't wanna go to that test, maybe she would be scared, even if she doesn't have cancer…."

FG2, woman, London.

Other negative aspects, such as the cost to the government or concerns that the test or 'diagnosis' could be wrong or inconclusive, were infrequently discussed.

3. Attitudes towards risk stratified management

Participants endorsed the risk stratified management approach and accepted the information that there would be clear options for women at each level of risk. No concerns were expressed about receiving different treatment based on level of risk.

…you will be able to find out what you have got and according to that you can prevent your, you know, things as well, if you want to go for like a screening or for a minor surgery or whatever it is, it's good to know what you have.

FG1, woman, London.

For peer review only - http://bmjopen.bmj.com/site/about/guidelines.xhtml

For peer review only

The screening component of risk-stratified management was generally endorsed, and participants felt that within their SA communities participation in current UK cancer screening programmes was gaining traction:

> *Well at least it's something for your health, good health*
>
> FG3, woman, London.
>
> *It's best to take a test, best to take a test not to get to that stage, isn't it?*
>
> FG7, man, Luton.

However, participants talked about there still being some within the SA community who do not accept cancer screening per se. A few participants, mostly men, suggested that some women may not see the need to attend screening in the absence of symptoms. Reluctance to attend screening was also attributed to fear and issues of body privacy and shyness. Women owned that exposing their body to "someone else" could be distressing, irrespective of whether the person was a health care professional:

> *What she's saying is that first, Muslims were a bit scared and they wouldn't get checks done. They thought that maybe someone else would see them...*
>
> FG4, woman, Luton.

Attitudes towards risk-reducing surgery were mixed. Whilst surgery was acceptable if it was deemed necessary by doctors, men and women said that women would not want surgery unless they had already had children and were of an older age.

> *It depends on age because, any lady who is 50 years up, that time is high risk, she needs to remove that, but 25 years, 30 years, any lady, she has still option for children, so she can't do that thing*
>
> FG2, woman, London.

For peer review only - http://bmjopen.bmj.com/site/about/guidelines.xhtml

Some men did not agree with risk-reducing surgery and believed that women would not accept it until cancer was diagnosed especially since 'high risk' did not mean the woman would definitely develop OC.

Until such a time that a person is diagnosed with cancer, I don't think they will have their ovaries removed.

FG5, man, Luton.

4. Family, culture and religion

The majority of women anticipated that their husbands and immediate families would support them if they had genetic testing for OC risk and most of the men also said that they would support their female family members if they wanted to be tested. Several women said they would discuss this beforehand with their husbands and families and/or would share the result.

And then obviously going back and discussing it with family what's come up as well.

FG3, woman, London.

Both men and women felt that some women would keep genetic testing and their result secret or 'confidential' from family and friends:

Some people may not want to share it with their family, want to keep it to themselves.

FG2, woman, London.

Some women who found the PROMISE programme personally acceptable, had concerns that younger women found to be at high risk for OC could have difficulties finding a husband due to the pro-natalist (promotion of human reproduction) nature of SA culture that favours the healthiest women. Others expressed concern that identifying someone as being at high risk or deciding to remove the ovaries could jeopardise existing marriages.

For peer review only - http://bmjopen.bmj.com/site/about/guidelines.xhtml

For peer review only

> *…if you get to our age then we would say yes. But for people like my daughter I would advise her not to. Because maybe the husband would leave the wife.*
>
> FG2, woman, London.

Some men indicated that they would not ask a potential partner about their cancer risk and that this would not influence their marital choice. Others spoke of the cultural importance of marriage and a woman's ability to bear children where removal of the ovaries would be a serious issue.

A few participants expressed the view that illnesses such as cancer are predestined and come from God. However, they did not suggest that this meant they would do nothing to prevent illness; instead religion was referred to as a coping resource.

> *...if I find out that I have got this problem; there will going to be ovarian cancer. I would thank God for giving me time to do what I want*
>
> FG1, woman, London.

5. Accessing services

Participants were eager for more information to be provided and for OC awareness campaigns within their communities.

> *They don't even know where it is…Where is the problem? How does it happen? It is very important that we give this information first.*
>
> FG4, woman, Luton.

A variety of methods to reach SA women with key OC prevention, earlier diagnosis and risk management messages were discussed including community based group sessions and campaigns in the local media targeted at those for whom English is not a first language. Several participants suggested that as English was not their first language or because they could not read English, they

For peer review only - http://bmjopen.bmj.com/site/about/guidelines.xhtml

would likely encounter difficulties in accessing genetic and screening services.

Both men and women indicated that, depending on the individual and situation, SA women would need or prefer to see a female healthcare professional, particularly if a physical examination was needed.

> *Mostly, the issues are about female doctors checking female things and male doctors checking males.*
>
> FG5, man, Luton.

Discussion

In this qualitative study, we identified a worrying lack of awareness of OC among SA women and men. Others have reported low awareness of cancer risk factors and symptoms amongst UK ethnic minority groups [25 26 27 28], although research has also shown a lack of ovarian cancer symptom awareness amongst the UK general population[28]. Irrespective of whether risk-stratified ovarian cancer management is offered to the public, improving awareness of OC amongst SAs is a health priority.

Some participants found it difficult to understand the brief presentation provided in the focus groups: it sometimes took several explanations to ensure that participants understood that genetic testing provides information on cancer risk rather than a diagnosis and that high risk did not mean that a person would definitely get cancer. As previously reported [11 12] participants tended to dichotomise risk as either high or low, with little discussion of intermediate risk. This underlines the need to develop optimal methods of conveying both the concept of OC risk and its meaning to the individual [26, 27]. The challenges of communicating risk estimates to the lay public are well documented and are particularly challenging [29-31] when information materials need to be acceptable to diverse populations.

For peer review only - http://bmjopen.bmj.com/site/about/guidelines.xhtml

Our main finding that attitudes toward genetic testing for OC risk and stratified management were mostly positive is consistent with other studies [32] but our study identified important cultural nuances. Participants maintained that personal genetic testing would not be viewed negatively from a religious standpoint and whilst a few referred to illness and death as predestined or from God, they indicated that it was still necessary to take action to maintain good health and, as in other research[33], religion was referred to as a coping strategy. Cancer fatalism was infrequently identified, but in this and other research [13], it was clear that the process of genetic testing and the receipt of a high risk result was anticipated by several participants to create heightened anxiety. Genetic testing for OC risk and stratified management may not be acceptable to all SA women, in particular younger women. Whilst the majority of women in this study indicated that they would accept a genetic test for OC risk if offered, many were already married and had children. Echoing research with UK Pakistanis about prenatal genetic testing [34 35] and UK SA women with breast cancer[36], some participants were concerned that illness or being identified as at high risk of OC could damage younger women's marriage prospects or cause marital problems. Participants acknowledged that not all SA women would discuss genetic testing or results with their family. Reluctance to discuss illness with family and friends due to taboo and perceived stigma was identified in the current study as well as in several other studies with SA participants [36 37], and could act as a barrier to the uptake of genetic testing.

Participants accepted the idea of stratified risk management, i.e. that there would be different management options for women with different levels of risk. With regard to the screening element of risk management, uptake of breast and cervical screening in the UK is lower amongst SA than White women[38]. Whilst the situation is slowly improving among SA women generally, change has not been significant for Muslim SA women [38]. Interestingly, in our study several groups discussed a positive cultural change in attitudes towards cancer screening and the majority reported attendance

For peer review only - http://bmjopen.bmj.com/site/about/guidelines.xhtml

at breast and/or cervical screening. In line with previous research [39-41], participants argued that lack of awareness, embarrassment and shyness were barriers to attending screening. Whilst OC screening with blood tests and ultrasound scans was seen as acceptable by most participants, the study did not explore the acceptability of *trans-vaginal* ultra-sound (the most commonly used scan to help detect OC), as this was beyond the scope of the study. However, as reported elsewhere [14 33], participants did have a preference for consultations with a gender-matched healthcare professional, particularly if a physical examination was needed.

Risk-reducing oophorectomy was seen as a particular dilemma, principally due to the importance placed on women's ability to bear children. Some male participants felt that it would be better to wait and see if a cancer develops, catch it at an early stage, and then have surgery. It may be that these men were inappropriately applying to OC their knowledge of how other cancers present and develop and this needs further investigation. However, apprehension about this surgery is not unique to SAs [42]. Our study highlights the need for sufficient information and support to be offered to SA women considering predictive genetic testing, and particularly for those with increased risk who will need to make risk management decisions.

This is the first study to explore UK SAs' perspectives on population-based genetic testing and risk stratified management for ovarian cancer, and includes participants with various levels of English language who are often not included in research. However, the opinions expressed by participants with regard to genetic testing and the PROMISE programme were based on brief information which was new to all, and related issues such as insurance and ethics were not spontaneously discussed. Furthermore, the current study did not inform patients that an increased risk of OC due to a *BRCA* gene mutation also indicates an increased risk of breast cancer, as this was beyond the scope of the study. The risk of breast cancer would further complicate decision-making as high risk patients would need to consider increased surveillance for breast cancer or risk-reducing mastectomy. Also,

For peer review only - http://bmjopen.bmj.com/site/about/guidelines.xhtml

both male focus groups were run by female facilitators; whilst participants did not express dissatisfaction with this, it may have influenced their responses.

Conclusions

Population-based risk assessment and stratified management may be acceptable to many SA men and women in the UK. Attitudes towards cancer screening were positive; however, opinions on risk-reducing surgery were mixed. The study highlights a need for tailored OC awareness campaigns within SA communities. To be inclusive, genetic testing and aftercare services should accommodate non-English speakers, offer appointments with a gender-matched healthcare professional, and offer patients support with their healthcare decisions.

For peer review only - http://bmjopen.bmj.com/site/about/guidelines.xhtml

Table 1. Sample demographics (n= 62)

	n (%)
**Gender**	
Female	49 (79.0)
Male	13 (21.0)
**Age**	
Mean years (range)	50.5 (22 - 82)
**Ethnic group**	
Bangladeshi	31 (50.0)
Indian	14 (22.6)
Pakistani	15 (24.2)
Other, Kashmiri	2 (3.2)
**Approx. years lived in the UK**	
Mean (range)	28.0 (2 - 49)
**First language***	
English	8 (12.9)
Bengali/Bangla	32 (51.6)
Gujarati	3 (4.8)
Hindi	5 (8.1)
Pahari	5 (8.1)
Punjabi	4 (6.5)
Sylheti	1 (1.6)
Urdu	15 (24.2)
Missing	1 (1.6)
**Religion**	
Hindu	7 (11.3)
Muslim	52 (83.9)
Sikh	3 (4.8)
**Marital status**	
Married/living with partner	44 (71.0)
Single/separated/divorced/widowed	17 (27.4)
Missing	1 (1.6)
**Employment**	
Full-time employment	4 (6.5)
Part-time employment	8 (12.9)
Homemaker	14 (22.6)
Retired	14 (22.6)

For peer review only - http://bmjopen.bmj.com/site/about/guidelines.xhtml

Disabled/too ill to work/ fulltime carer	3 (4.8)
Unemployed	19 (30.6)
**Education**	
Degree or higher	12 (19.4)
Qualification below degree level	19 (30.64)
Still studying	1 (1.8)
Other	11 (17.7)
No formal qualifications	19 (30.6)
**Attended screening**	
Amongst female participants (Breast or cervical screening or FOBT)	39 (79.6)
Amongst male participants (FOBT)	2 (15.4)
**Cancer within social network**	
Yes	29 (46.8)
No/Not sure/Prefer not to say	33 (53.2)
**Personal cancer diagnosis**	
Yes	3 (4.8)
No/Not sure/Prefer not to say	59 (95.2)

*Some participants had more than one first language.

For peer review

For peer review only - http://bmjopen.bmj.com/site/about/guidelines.xhtml

Acknowledgements

This work was carried out at UCLH/UCL within the Cancer Theme of the NIHR UCLH/UCL Comprehensive Biomedical Research Centre supported by the UK Department of Health for the PROMISE study team.

We thank all the women and men who took part in the focus groups and all the centres and community group leaders who helped to recruit participants and distribute posters for the study.

Disclosure of interests

All authors declare that they have no competing interests.

Contribution to authorship

Authors KH, NA, LF, LS, JW, SS and AL contributed to the design of the study including refinement of the discussion guide. AL was overall responsible for the delivery of the project. NA and KH recruited participants and conducted the 7 focus group discussions. KH analysed the data and NA and AL checked the data for any divergent cases. Independent researcher SG performed coding checks on a proportion of the data. Authors KH, NA, LF, LS, JW, SS, SG, and AL contributed to interpretation of the results. KH drafted the manuscript and authors KH, NA, LF, LS, JW, SS, SG and AL critically reviewed and approved the manuscript.

Details of ethics approval

Approval to conduct the study was granted by the UCL Research Ethics Committee (project ID: 8053/003).

Funding

This work was supported by The Eve Appeal (509050) and Cancer Research UK (C1005/A12677). The

For peer review only - http://bmjopen.bmj.com/site/about/guidelines.xhtml

funders had no role in the study design; collection, management, analysis, or interpretation of data; writing of the report; or the decision to submit the report for publication.

Data sharing statement

Anonymised qualitative data is available on request.

Supplementary files

COREQ checklist

Focus Group Discussion Guide (Women)

Focus group Discussion Guide (Men)

Focus Group Information slides

For peer review only - http://bmjopen.bmj.com/site/about/guidelines.xhtml

References

1. Cancer Research UK. Ovarian cancer incidence by UK country. 2016. http://www.cancerresearchuk.org/health-professional/cancer-statistics/statistics-by-cancer-type/ovarian-cancer/incidence#heading-Zero.
2. The Office for National Statistics. Cancer survival by stage at diagnosis for England (experimental statistics): Adults diagnosed 2012, 2013 and 2014 and followed up to 2015. 2016. https://www.ons.gov.uk/peoplepopulationandcommunity/healthandsocialcare/conditionsanddiseases/bulletins/cancersurvivalbystageatdiagnosisforenglandexperimentalstatistics/adultsdiagnosed20122013and2014andfollowedupto2015.
3. Ovacome. http://www.ovacome.org.uk/information/screening/.
4. Menon U, Gentry-Maharaj A, Hallett R, et al. Sensitivity and specificity of multimodal and ultrasound screening for ovarian cancer, and stage distribution of detected cancers: results of the prevalence screen of the UK Collaborative Trial of Ovarian Cancer Screening (UKCTOCS). *The Lancet Oncology* 2009;10:327-40.
5. Rosenthal AN, Fraser LS, Philpott S, et al. Evidence of stage shift in women diagnosed with ovarian cancer during phase II of the United Kingdom Familial Ovarian Cancer Screening Study. *Journal of Clinical Oncology* 2017:JCO-2016.
6. Jacobs I, Menon U, Ryan A, et al. Ovarian cancer screening and mortality in the UK Collaborative Trial of Ovarian Cancer Screening (UKCTOCS): a randomised controlled trial. *The Lancet* 2016;387(10022):945-56.
7. Antoniou A, Pharoah PD, Narod S, et al. Average risks of breast and ovarian cancer associated with BRCA1 or BRCA2 mutations detected in case series unselected for family history: a combined analysis of 22 studies. *The American Journal of Human Genetics* 2003;72(5):1117-30.
8. Manchanda R, Jacobs I. Genetic screening for gynecological cancer: where are we heading? *Future Oncology* 2015;12(2):207–20.
9. Burton H, Chowdhury S, Dent T, et al. Public health implications from COGS and potential for risk stratification and screening. *Nature Genetics* 2013;45(4):349.
10. Davies SC. Annual report of the Chief Medical Officer 2016, Generation genome. In: Health Do, ed. London, 2017.
11. Meisel SF, Side L, Fraser L, et al. Population-based, risk-stratified genetic testing for ovarian cancer risk: A focus group study. *Public Health Genomics* 2013;16:184-91.
12. Rahman B, Meisel SF, Fraser L, et al. Population-based genetic risk prediction and stratification for ovarian cancer: views from women at high risk. *Familial Cancer* 2015;14(1):135-44.
13. Hann KEJ, Freeman M, Fraser L, et al. Awareness, knowledge, perceptions, and attitudes towards genetic testing for cancer risk among ethnic minority groups: a systematic review. *BMC Public Health* 2017;17(1):503.
14. Atkin K, Ali N, Chu CE. The politics of difference? Providing a cancer genetics service in a culturally and linguistically diverse society. *Diversity in Health and Care* 2009;6(3):149–57.
15. Genetic Alliance UK. Identifying family risk of cancer: Why is this more difficult for ethnic minority communities and what would help? London, 2012.
16. The Office for National Statistics. Ethnicity and national identity in England and Wales: 2011. 2011. http://www.ons.gov.uk/peoplepopulationandcommunity/culturalidentity/ethnicity/articles/ethnicityandnationalidentityinenglandandwales/2012-12-11.
17. Grewal S, Bottorff JL, Hilton BA. The influence of family on immigrant South Asian women's health. *Journal of Family Nursing* 2005;11(3):242-63.
18. Kushniryk A, Orlov S, Wertz E. Health and culture: Factors influencing immigrant women's health beliefs and health behaviour. *Online Journal of Communication and Media Technologies* 2017;7(1):104-25.
19. Burr V. *An introduction to social constructionism*. London: Routledge, 1995.

For peer review only - http://bmjopen.bmj.com/site/about/guidelines.xhtml

20. Kitzinger J. Qualitative research. Introducing focus groups. *BMJ* 1995;311:299-302.
21. Morse JM. Determining sample size. *Qualitative Health Research* 2000;10(1):3-5.
22. Huo D, Olopade OI. Genetic testing in diverse populations: are researchers doing enough to get out the correct message? . *The Journal of the American Medical Association* 2007;298(24):2910-11.
23. Braun V, Clarke V. Using thematic analysis in psychology. *Qualitative Research in Psychology* 2006;3(3):77-101.
24. Tong A, Sainsbury P, Craig J. Consolidated criteria for reporting qualitative research (COREQ): a 32-item checklist for interviews and focus groups. *Int J Qual Health Care* 2007;19(6):349-57.
25. Waller J, Robb K, Stubbings S, et al. Awareness of cancer symptoms and anticipated help seeking among ethnic minority groups in England. *British Journal of Cancer* 2009;101:S24-S30.
26. Marlow LAV, Robb KA, Simon AE, et al. Awareness of cancer risk factors among ethnic minority groups in England. *Public Health* 2012;126(8):702-09.
27. Fallowfield L, Fleissig A, Barrett J, et al. Awareness of ovarian cancer risk factors, beliefs and attitudes towards screening: baseline survey of 21 715 women participating in the UK Collaborative Trial of Ovarian Cancer Screening. . *British Journal of Cancer* 2010;103(4):454-61.
28. Low EL, Waller J, Menon U, et al. Ovarian cancer symptom awareness and anticipated time to help-seeking for symptoms among UK women. *Journal of Family Planning and Reproductive Health Care* 2013;39:163-71.
29. Janssen E, van Osch L, de Vries H, et al. The influence of narrative risk communication on feelings of cancer risk. . *British Journal of Health Psychology* 2013;18(2):407-19.
30. Han PKJ, Klein WMP, Lehman T, et al. Communication of uncertainty regarding individualized cancer risk estimates: Effects and influential factors. *Medical Decision Making* 2011;31:354-66.
31. Peters E, McCaul KD, Stefanek M, et al. A heuristics approach to understanding cancer risk perception: Contributions from judgment and decision-making research. *Annals of Behavioral Medicine* 2006;31(1):45-52.
32. Lerman C, Daly M, Masny A, et al. Attitudes about genetic testing for breast-ovariancancer susceptibility. *Journal of Clinical Oncology* 1994;12(4):843-50.
33. Thomas VN, Saleem T, Abraham R. Barriers to effective uptake of cancer screening among black and minority ethnic groups. *International Journal of Palliative Nursing* 2005;11(11):562-71.
34. Shaw A, Hurst JA. 'I don't see any point in telling them': attitudes to sharing genetic information in the family and carrier testing of relatives among British Pakistani adults referred to a genetics clinic. *Ethnicity & Health* 2009;14(2):205-24.
35. Darr A, Small N, Ahmad WI, et al. Examining the family-centred approach to genetic testing and counselling among UK Pakistanis: a community perspective. *Journal of Community Genetics* 2013;4(1):49-57.
36. Karbani G, Lim JNW, Hewison J, et al. Culture, attitude and knowledge about breast cancer and preventive measures: a qualitative study of South Asian breast cancer patients in the UK. *Asian Pacific Journal of Cancer Prevention* 2011;12(6):1619-26.
37. Randhawa G, Owens A. The meanings of cancer and perceptions of cancer services among South Asians in Luton. *British Journal of Cancer* 2004;91(1):62-68.
38. Szczepura A, Price C, Gumber A. Breast and bowel cancer screening uptake patterns over 15 years for UK south Asian ethnic minority populations, corrected for differences in socio-demographic characteristics. *BMC Public Health* 2008;8(1):346.
39. Marlow LAV, Waller J, Wardle J. Barriers to cervical cancer screening among ethnic minority women: a qualitative study. *Journal of Family Planning and Reproductive Health Care* 2015;0:1-7.

For peer review only - http://bmjopen.bmj.com/site/about/guidelines.xhtml

40. Crawford J, Ahmad F, Beaton D, et al. Cancer screening behaviours among South Asian immigrants in the UK, US and Canada: a scoping study. *Health and Social Care in the Community* 2016;24(2):123–53.
41. Robb K, Wardle J, Stubbings S, et al. Ethnic disparities in knowledge of cancer screening programmes in the UK. *Journal of Medical Screening* 2010;17(3):125-31.
42. Tong A, Kelly S, Nusbaum R, et al. Intentions for risk-reducing surgery among high-risk women referred for BRCA1/BRCA2 genetic counseling. *Psycho-Oncology* 2015;24:33–39.

For peer review only

For peer review only - http://bmjopen.bmj.com/site/about/guidelines.xhtml

Title: Attitudes towards a programme of risk assessment and stratified management for ovarian cancer: A focus group study of UK South Asians' perspectives

Focus Group Discussion Guide (Women)

1. Introduction

- Welcome and thanks for taking part
- Introduction and purpose of the study
- Confidentiality (agree that the discussion should be confidential among respondents)
- Timing (up to 1 ½ hours)
- Reminder of audio-recording
- Anonymity in report writing etc.
- Ground rules – respect different opinions, keep mobile phones off or on silent.
- No right/wrong answers – hoping for a range of views
- Participants introduce themselves

2. General awareness/attitudes towards ovarian cancer risk

I would like to start by discussing what you know about ovarian cancer and what you think about your ovarian cancer risk

- What do you know about ovarian cancer?
  - Prompts: What do you know about how common ovarian cancer is/symptoms/ causes/ risk factors/reducing risk?
- What do you think about your own risk of ovarian cancer?
  - Prompt: Do you think your chances of developing ovarian cancer are the same as the rest of the UK population or higher or lower?

***Focus group participants presented with slides on genetic risk.**

3. Opinions on genetic information

We are working on a project where we will be inviting women to have genetic testing for ovarian cancer risk, regardless of whether they have a family history of cancer.

- Does genetic cancer risk information make sense to you?
  - What did you know about genetic testing for cancer risk before coming to this discussion today?
  - Does the link between genetics and risk of ovarian cancer make sense?
- How would you feel about having genetic testing and receiving test results on ovarian cancer risk?
  - Prompt: Pros/cons of testing?
  - Prompt: Pros/cons of finding out test result?
- What might influence your decision to have genetic testing for ovarian cancer risk if it were offered?
  - Prompt: Friends/ Family/ Religion/ Culture/ Access/ Practical issues/ Concerns?
- How do you think your family and friends would view your decision of having (or not having) genetic testing for risk of ovarian cancer?
  - Prompt: Husband/ Sisters/ Parents/ Children/ Close female friends?

For peer review only

4. Opinions of risk stratification approach and possible risk management options

In our project, women will be told whether they have a low, intermediate or high risk for ovarian cancer, based on a combination of their genetic risk and other risk factors. Identifying other risk factors is done using questions about family history, lifestyle and health information. We expect that most of the women will have a low risk result, fewer will have an intermediate risk, and fewer still will have a high risk. Being at low risk would not mean that the women have no risk of developing ovarian cancer.

Depending on their risk level, women would be offered different interventions. Women at low risk would receive information telling them that they are low risk and don't need further monitoring, they would also be given information about symptoms of ovarian cancer to be aware of. Women at intermediate risk would be offered screening every four months involving a blood test and a yearly ultrasound scan. Preventative surgery, involving the removal of the ovaries, would be discussed as an option depending on the characteristics of the woman (such as age, their family history of cancer etc). For high risk women, preventative surgery would be offered as a main option, but screening would also be discussed with these patients.

We are wondering whether women might be interested in taking part in this research and how women might feel about it if it were broadened out into a general population service.

- What do you think about this idea?
    - Prompt: Pros and cons?
    - Prompt: Culturally acceptable? Religiously acceptable?
    - Prompt: What do you think about the idea of offering different care to women depending on their level of risk?
    - Prompt: What do you think about the risk management options?
- How would you feel if you were in the low/intermediate/high risk management group?
    - Prompt: Pros and cons?
    - Prompt: How might it impact you/ your family?
- How would you feel about taking part in this project if you were invited?
    - Prompt: Would you be interested in taking part?

Much of what we know about ovarian cancer risk due to inherited genetics comes from research with white women of European descent. Whilst there is no evidence to suggest that this risk differs between ethnic groups, we don't know for certain. It is possible that the estimated cancer risk based on previous research may be less accurate for other ethnic groups.

- How might this information impact on whether or not you would agree to have your cancer risk estimated from genetic and other information/ impact on taking part in the trial if invited?

- How might this programme be provided in a way that you would find acceptable and accessible?
    - Prompt: What would be important to you about how the service is provided?
    - Prompt: Barriers/facilitators?

5. Final comments

- Is there anything else that you'd like to talk about that you think might be relevant?

6. Debrief and thank participants

For peer review only

Title: Attitudes towards a programme of risk assessment and stratified management for ovarian cancer: A focus group study of UK South Asians' perspectives

Focus Group Discussion Guide (Men)

1. Introduction

- Welcome and thanks for taking part
- Introduction and purpose of the study
- Confidentiality (agree that the discussion should be confidential among respondents)
- Timing (up to 1 ½ hours)
- Reminder of audio-recording
- Anonymity in report writing etc.
- Ground rules – respect different opinions, keep mobile phones off or on silent
- No right/wrong answers – hoping for a range of views
- Participants introduce themselves

2. General awareness/attitudes towards ovarian cancer risk

- I'd like to start by discussing what you know about ovarian cancer?
    - Prompts: What do you know about how common ovarian cancer is/symptoms/ causes/ risk factors/reducing risk?

***Focus group participants presented with slides on genetic risk.**

3. Opinions on genetic information

We are working on a project where we will be inviting women to have genetic testing for ovarian cancer risk, regardless of whether they have a family history of cancer.

- Does genetic cancer risk information make sense to you?
    - What did you know about genetic testing for cancer risk before coming to this discussion today?
    - Does the link between genetics and risk of ovarian cancer make sense?
- How would you feel about female members of your family (your wife/ sister/ mother/ daughter) having genetic testing and receiving test results on ovarian cancer risk?
    - Prompt: pros/cons? Culturally acceptable? Religiously acceptable?
    - Prompt: What impact could it have on you/your family?

4. Opinions of risk stratification approach and possible risk management options

In our project, women will be told whether they have a low, intermediate or high risk for ovarian cancer, based on a combination of their genetic risk and other risk factors. Identifying other risk factors is done using questions about family history, lifestyle and health information. We expect that most of the women will have a low risk result, fewer will have an intermediate risk, and fewer still will have a high risk. Being at low risk would not mean that the women have no risk of developing ovarian cancer.

Depending on their risk level, women would be offered different interventions. Women at low risk would receive information telling them that they are low risk and don't need further monitoring, they would also be given information about symptoms of ovarian cancer to be aware of. Women at intermediate risk would be offered screening every four months involving a blood test to check for levels of a biomarker and a yearly ultrasound scan. Preventative surgery, involving the removal of the

For peer review only

ovaries, would be discussed as an option depending on the characteristics of the woman (such as age, their family history of cancer etc). For high risk women, preventative surgery would be offered as a primary option, but screening would also be discussed with these patients.

- What do you think about this idea?
    - Prompt: Pros and cons?
    - Prompt: Culturally acceptable? Religiously acceptable?
    - Prompt: What do you think about the idea of offering different care/options to women depending on their level of risk?
    - Prompt: What do you think about the risk management options (information/screening/surgery)?
    - Thinking about wife/mother/sister: what impact do you think a high/ intermediate/ low risk would have for them/the family?

Much of what we know about ovarian cancer risk due to inherited genetics comes from research with white women of European descent. Whilst there is no evidence to suggest that this risk differs between ethnic groups, we don't know for certain. It is possible that the estimated cancer risk based on previous research may be less accurate for other ethnic groups.

- How might this information impact on whether or not you would support or encourage female family members' decision to have their cancer risk estimated from genetic and other information?

- How might this programme of genetic testing and risk management be provided in a way that would be acceptable and accessible?
    - What would be important about how the service is provided?
    - Barriers/facilitators?

<u>5. Final comments</u>

- Is there anything else that you'd like to talk about that you think might be relevant?

<u>6. Debrief and thank participants</u>

For peer review only

Ovarian cancer risk

- The lifetime risk of a women in the UK getting ovarian cancer is about 2%.
- This means around 1 in 50 women will develop ovarian cancer at some point in their life.

For peer review only - http://bmjopen.bmj.com/site/about/guidelines.xhtml

Ovarian cancer risk

- We don’t know the cause of most ovarian cancers.
- We know some of the ‘**risk factors**’ – these are things that may increase the chances of developing cancer.
- Having a cancer risk factor doesn’t mean that a person will definitely get cancer – just as not having it doesn’t mean that they won’t.

Genes

- Genes carry the biological information passed from parent to child.
- Some genes are known to increase the risk of ovarian cancer.
- Changes (mutations) in certain genes are known to increase the risk of cancer.

UCL

Ovarian cancer genes

- Mutations in two genes – called BRCA1 and BRCA2 – increase the risk of ovarian cancer.
- BRCA1 and BRCA2 mutations are very rare but family members who inherit them have a much greater risk of ovarian cancer.
- Scientists can also identify other relevant gene mutations involved.

Testing for genetic risk

- It is possible to test for BRCA1 and BRCA2 gene mutations.
- Genetic testing involves a blood test.
- Genetic material (DNA) is taken from the blood cells to test for mutations.
- At present genetic testing for ovarian cancer is not available on the NHS except for women with a strong family history of cancer.

For peer review only - http://bmjopen.bmj.com/site/about/guidelines.xhtml

Importance of family history of cancer

- Families with a strong 'family history' of ovarian cancer are more likely to carry the gene mutations.
- A strong family history means:
  - Two close relatives (mother, sister, daughter) with ovarian cancer
  - One close relative with ovarian cancer, and, on the same side of the family:
    - One close relative who had breast cancer before age 50
    - Two close relatives who had breast cancer before age 60
    - Three close relatives who had bowel or womb (uterus) cancer

PROMISE research programme

- Genetic testing + personal and lifestyle information = risk
- Women who agree to this will be grouped as being at **high**, **intermediate** or **low** risk for ovarian cancer.

For peer review only - http://bmjopen.bmj.com/site/about/guidelines.xhtml

UCL

PROMISE continued…

- The doctor would then discuss different risk management options:

- ➢ Low risk: symptom awareness information.
- ➢ Intermediate risk: screening, or surgery depending on age.
- ➢ High risk: surgery, or screening if not ready to have surgery.

COREQ (COnsolidated criteria for REporting Qualitative research) Checklist

A checklist of items that should be included in reports of qualitative research. You must report the page number in your manuscript where you consider each of the items listed in this checklist. If you have not included this information, either revise your manuscript accordingly before submitting or note N/A.

Topic	Item No.	Guide Questions/Description	Reported on Page No.
**Domain 1: Research team and reflexivity**			
Personal characteristics			
Interviewer/facilitator	1	Which author/s conducted the interview or focus group?	
Credentials	2	What were the researcher's credentials? E.g. PhD, MD	
Occupation	3	What was their occupation at the time of the study?	
Gender	4	Was the researcher male or female?	
Experience and training	5	What experience or training did the researcher have?	
Relationship with participants			
Relationship established	6	Was a relationship established prior to study commencement?	
Participant knowledge of the interviewer	7	What did the participants know about the researcher? e.g. personal goals, reasons for doing the research	
Interviewer characteristics	8	What characteristics were reported about the inter viewer/facilitator? e.g. Bias, assumptions, reasons and interests in the research topic	
**Domain 2: Study design**			
Theoretical framework			
Methodological orientation and Theory	9	What methodological orientation was stated to underpin the study? e.g. grounded theory, discourse analysis, ethnography, phenomenology, content analysis	
Participant selection			
Sampling	10	How were participants selected? e.g. purposive, convenience, consecutive, snowball	
Method of approach	11	How were participants approached? e.g. face-to-face, telephone, mail, email	
Sample size	12	How many participants were in the study?	
Non-participation	13	How many people refused to participate or dropped out? Reasons?	
Setting			
Setting of data collection	14	Where was the data collected? e.g. home, clinic, workplace	
Presence of non-participants	15	Was anyone else present besides the participants and researchers?	
Description of sample	16	What are the important characteristics of the sample? e.g. demographic data, date	
Data collection			
Interview guide	17	Were questions, prompts, guides provided by the authors? Was it pilot tested?	
Repeat interviews	18	Were repeat inter views carried out? If yes, how many?	
Audio/visual recording	19	Did the research use audio or visual recording to collect the data?	
Field notes	20	Were field notes made during and/or after the inter view or focus group?	
Duration	21	What was the duration of the inter views or focus group?	
Data saturation	22	Was data saturation discussed?	
Transcripts returned	23	Were transcripts returned to participants for comment and/or	

For peer review only

Topic	Item No.	Guide Questions/Description	Reported on Page No.
		correction?	
**Domain 3: analysis and findings**			
Data analysis			
Number of data coders	24	How many data coders coded the data?	
Description of the coding tree	25	Did authors provide a description of the coding tree?	
Derivation of themes	26	Were themes identified in advance or derived from the data?	
Software	27	What software, if applicable, was used to manage the data?	
Participant checking	28	Did participants provide feedback on the findings?	
Reporting			
Quotations presented	29	Were participant quotations presented to illustrate the themes/findings? Was each quotation identified? e.g. participant number	
Data and findings consistent	30	Was there consistency between the data presented and the findings?	
Clarity of major themes	31	Were major themes clearly presented in the findings?	
Clarity of minor themes	32	Is there a description of diverse cases or discussion of minor themes?	

Developed from: Tong A, Sainsbury P, Craig J. Consolidated criteria for reporting qualitative research (COREQ): a 32-item checklist for interviews and focus groups. *International Journal for Quality in Health Care*. 2007. Volume 19, Number 6: pp. 349 – 357

Once you have completed this checklist, please save a copy and upload it as part of your submission. DO NOT include this checklist as part of the main manuscript document. It must be uploaded as a separate file.

For peer review only
